# Aortic disease-linked mutations reveal unexpected convergent allosteric mechanisms of protein kinase G misregulation

**DOI:** 10.1126/sciadv.aeg1640

**Published:** 2026-09-02

**Authors:** Karla Martinez Pomier, Leopold Jahn, Bryan VanSchouwen, Hebatallah Mohamed, Madoka Akimoto, Sun Woo Jung, Daniela Bertinetti, Johannes Mehringer, Leonardo Della Libera, Friederike Cuello, Friedrich W. Herberg, Giuseppe Melacini

**Affiliations:** ^1^ Department of Chemistry and Chemical Biology, McMaster University, Hamilton, ON, Canada.; ^2^ Institute for Biology, Department of Biochemistry, University of Kassel, Kassel, Germany.; ^3^ Kurt Schwabe Institute for Sensor Technologies, Waldheim, Germany.; ^4^ Institute of Experimental Pharmacology and Toxicology, University Medical Center Hamburg-Eppendorf, Hamburg, Germany.; ^5^ DZHK (German Center for Cardiovascular Research), Partner site North, University Medical Center Hamburg-Eppendorf, Hamburg, Germany.; ^6^ Department of Biochemistry and Biomedical Sciences, McMaster University, Hamilton, ON, Canada.

## Abstract

Thoracic aortic aneurysms and dissections (TAAD) are life-threatening conditions linked to gain-of-function mutations in cGMP-dependent protein kinase I (PKG I), a central regulator of vascular smooth muscle signaling. Among these, substitutions at Val^234^ have been associated with kinase overactivation and early-onset disease, despite this residue being distal from the active site and cGMP binding regions. Using NMR, molecular dynamics simulations, and complementary kinase and binding assays, we show that Val^234^ acts as a crucial allosteric hub of PKG autoinhibition. TAAD-associated variants at Val^234^ disrupt PKG regulation through two distinct yet complementary allosteric mechanisms: by biasing the kinase toward active conformations that increase sensitivity to cGMP and by decreasing the folding stability of the regulatory domain, thereby weakening inhibitory contacts independently of cGMP control. Despite these differences, both mechanisms result in excessive PKG signaling at basal and intermediate cGMP levels, while maximal activity remains unaltered. Together, these findings explain how distal mutations can unpredictably rewire kinase allostery and drive pathogenic vascular signaling.

## INTRODUCTION

Thoracic aortic aneurysms are characterized by the dilation and progressive weakening of the ascending aorta and can lead to life-threatening dissections or rupture of the aortic wall (*1*). While most thoracic aortic aneurysm and dissection (TAAD) cases arise from uncontrolled hypertension, a substantial subset (∼20%) originates from familial autosomal-dominant mutations involving genes that regulate smooth muscle function (*2*, *3*). For example, pathogenic mutations in the *PRKG1* gene, encoding type I cyclic guanosine 3′,5′-monophosphate (cGMP)–dependent protein kinase (PKG I), have emerged as critical molecular drivers of TAAD (*3*, *4*). The *PRKG1* gene encodes for two isoforms (PKG Iα and PKG Iβ) that differ in their first ∼100 amino acids and are both expressed in the cardiovascular system (*5*–*7*).

**Fig. 1.**
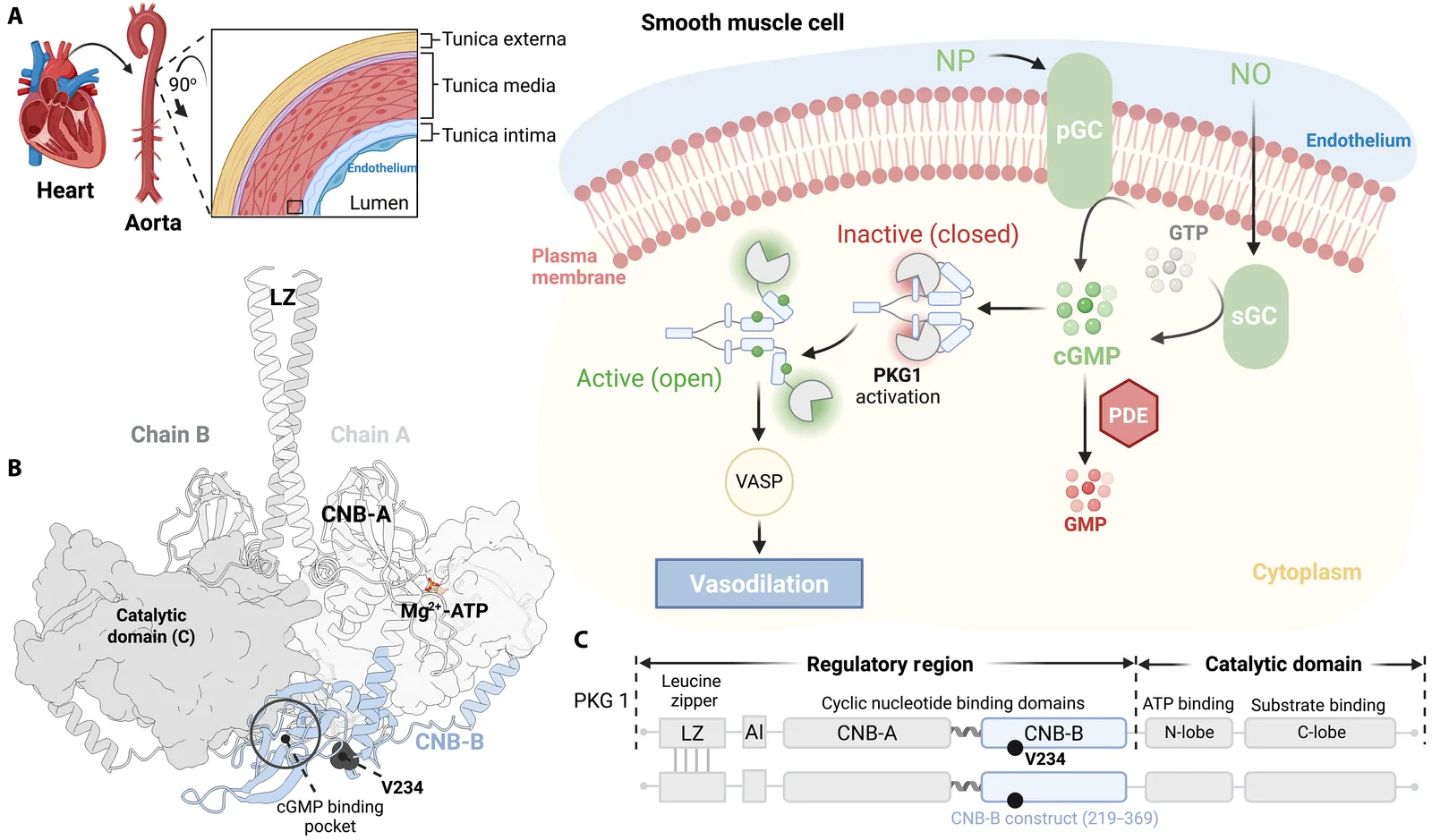
PKG I structure and function. (**A**) Mechanism of PKG I activation in an aortic smooth muscle cell. NP indicates natriuretic peptide; NO, nitric oxide; pGC, particulate guanylyl cyclase; sGC, soluble guanylyl cyclase; PDE, phosphodiesterase; VASP, vasodilator-stimulated phosphoprotein. (**B**) AlphaFold3 (*13*) predicted structure of the autoinhibited conformation of PKG Iβ in its dimeric form. Distinctive features are labeled, including the leucine zipper (LZ), the catalytic domain (C), the regulatory domains (CNB-A and CNB-B), the autoinhibitory (AI) site, and the ATP/Mg^2+^ binding site in the C-domain. Position V234 is indicated in the structure as well as the cGMP binding pocket of CNB-B. (**C**) Domain structure of PKG I, where the isolated domain construct (CNB-B) of our study is highlighted in blue, as well as in (B). Position V234 is represented by a black dot. Created in BioRender. Martinez Pomier, K. (2026) https://BioRender.com/p15rg13.

PKG I is a homodimeric serine/threonine kinase that controls vascular tone and cytoskeletal organization through cGMP-regulated allostery (Fig. 1A) (*5*, *6*, *8*–*12*). Each monomer contains a regulatory region with an N-terminal leucine zipper for dimerization and anchoring, followed by an autoinhibitory segment and the tandem cGMP-binding domains (CNB-A and CNB-B), which together occlude access to the kinase active site in the C-terminal catalytic domain (C-domain) (Fig. 1, B and C). In the absence of cGMP, contacts between the regulatory region and the C-domain maintain PKG I in a closed, inactive state; binding of cGMP relieves this inhibition by stabilizing a more open topology that unleashes the protein kinase activity (Fig. 1, A and B) (*8*, *13*). Phosphodiesterases terminate the signal by hydrolyzing cGMP, which reduces cGMP concentration to basal levels and brings PKG back to its inactive conformation (Fig. 1A) (*14*).

Gain-of-function mutations that constitutively activate PKG I and/or sensitize PKG I to cGMP result, for example, in increased phosphorylation of the myosin regulatory light chain and weakened vascular smooth muscle contraction, ultimately leading to enhanced susceptibility to aneurysm formation (*3*, *4*). This is the case for the constitutively active R177Q variant of PKG Iα (*4*). In addition, we recently reported that a valine-to-isoleucine substitution in CNB-B (i.e., V219I for the PKG isoform Iα or V234I for the PKG Iβ isoform) is strongly linked to TAAD in young patients (*15*). V234I produces enlarged, highly deformable cells with disrupted actin organization and extracellular matrix formation and displays constitutive kinase activity with elevated cGMP affinity (*15*).

At the structural level, given that V234 is distal from both the cGMP-binding pocket and the inhibitory interface between CNB-B and the catalytic domain (Fig. 1B), we hypothesize that the increased sensitivity to cGMP of V234I and its constitutive activation are the result of allosteric perturbations elicited by this mutant. V234 is part of a highly conserved structural motif of CNB-B, the N3A motif (fig. S1, A and B), which plays a prominent role in the allosteric regulation of cyclic nucleotide–regulated kinases (*16*, *17*).

To test our hypothesis and map the allosteric networks controlled by V234, we investigated the V234I variant by integrating kinase assays, isothermal titration calorimetry (ITC), switchSENSE experiments, analytical size exclusion chromatography (SEC), nuclear magnetic resonance (NMR), and molecular dynamics (MD) simulations. To further understand how the V234 side chain allosterically controls PKG activation, we extended our integrated experimental approach to other V234 variants with different side chain sizes. To this end, we searched three major clinical variant repositories: the Clinical Variants database (ClinVar), the Genome Aggregation Database (gnomAD), and the Catalogue of Somatic Mutations in Cancer (COSMIC). In addition to V234I, two other substitutions at this position were identified (V234F and V234A). V234F is reported in gnomAD and ClinVar in association with TAAD, whereas V234A is cataloged in COSMIC as a cancer-associated variant. These variants introduce either a bulkier or smaller side chain than valine, enabling us to probe the allosteric role of V234 in the context of PKG regulation (fig. S1, D to G).

The comparative analysis of V234I, V234F, and V234A, through the lens of our integrated functional and biophysical characterizations, revealed two distinct allosteric mechanisms by which V234 variants modulate PKG activity: (i) sampling, in the absence of cGMP, a conformation that resembles a partially active intermediate with apparent enhanced affinity for cGMP; (ii) the decrease of local and global folding stability of CNB-B that destabilizes crucial inhibitory contacts with the catalytic domain. Both mechanisms can work simultaneously, but one can prevail over the other depending on the specific PKG I variant.

## RESULTS

### V234 variants alter the cGMP-dependent activation of PKG Iβ

**Fig. 2.**
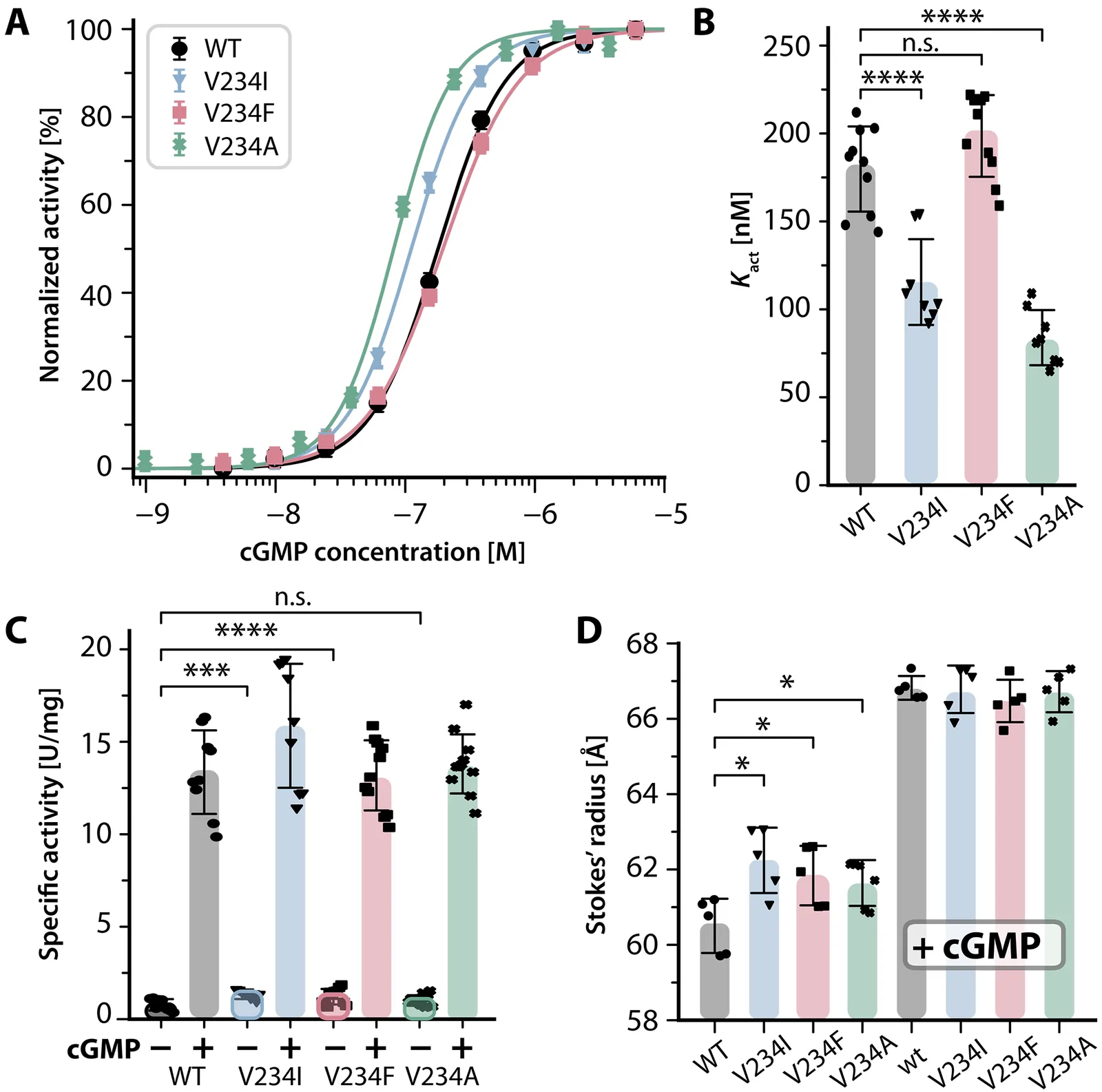
Activation of PKG Iβ variants in response to cGMP and VASPtide. Kinase activity was measured with a coupled spectrophotometric assay using a peptide substrate (VASPtide). (**A**) Normalized PKG Iβ activities were plotted against different cGMP concentrations. Basal activities (samples without cGMP) were set to 0%, while respective maximal activities were set to 100%. (**B**) Activation constants corresponding to 50% of kinase activity were deduced from activation curves (A). (**C**) Specific activities were measured under basal conditions (−cGMP) and in the presence of 5 μM cGMP (+cGMP). (**D**) Stokes’ radii were determined by SEC for PKG Iβ WT and variants in the absence of cGMP (left) and the presence of 25 μM cGMP (right). Data are means ± SD of at least three independent experiments. Statistical significance was tested with an unpaired Student’s *t* test against the WT dataset (n.s., nonsignificant; *P < 0.05, ***P < 0.001, and ****P < 0.0001).

As a first step toward understanding the activation mechanism of the three PKG Iβ V234 variants (V234I/F/A), we conducted kinase activity assays in the absence and presence of cGMP, using the substrate VASPtide, a peptide derived from the physiological PKG I target vasodilator-stimulated phosphoprotein (VASP) (*18*). Our kinase assays focused on the measurement of activation constants (*K*_act_) and basal kinase activities, which respectively report on cGMP-dependent allosteric regulation and the extent of autoinhibition under resting conditions. The resulting sigmoidal dose-response profiles (Fig. 2A) were used to determine activation constants *K*_act_ (Fig. 2B). Both V234I and V234A exhibited enhanced cGMP sensitivity compared to the wild type (WT), whereas the cGMP dependence of V234F activation remained similar to WT (Fig. 2, A and B). We also investigated the effect of these variants on the basal and the maximum activities of PKG Iβ. The V234I and V234F variants showed significantly increased basal activities compared to WT, while V234A preserved WT-like basal activity (Fig. 2C). All variants could be activated by cGMP to a similar maximum extent without significant differences from the WT (Fig. 2C and table S1).

To shed light on the distinct activation patterns of the V234 variants and the effect of cGMP on the overall PKG topology, we used analytical SEC. Measurements of cGMP-free PKG apoenzyme (apo) revealed a Stokes’ radius of 60.5 ± 0.8 Å for WT, while all variants showed minimal but significantly larger Stokes’ radii between 61.6 and 62.2 Å (Fig. 2D, left) (table S2). Measurements in the presence of cGMP showed a pronounced increase in Stokes’ radii for all proteins. The absolute values measured in the presence of cGMP were comparable for WT and the variants, ranging between 66.5 and 66.9 Å (Fig. 2D, right). Therefore, WT showed the largest increase (+6.4 Å), whereas the variants increased only by 4.6 Å (V234I), 4.7 Å (V234F), and 5.1 Å (V234A), suggesting a less compact apo/inactive topology.

### The switchSENSE technology confirms changes in conformational dynamics of PKG Iβ V234 variants

**Fig. 3.**
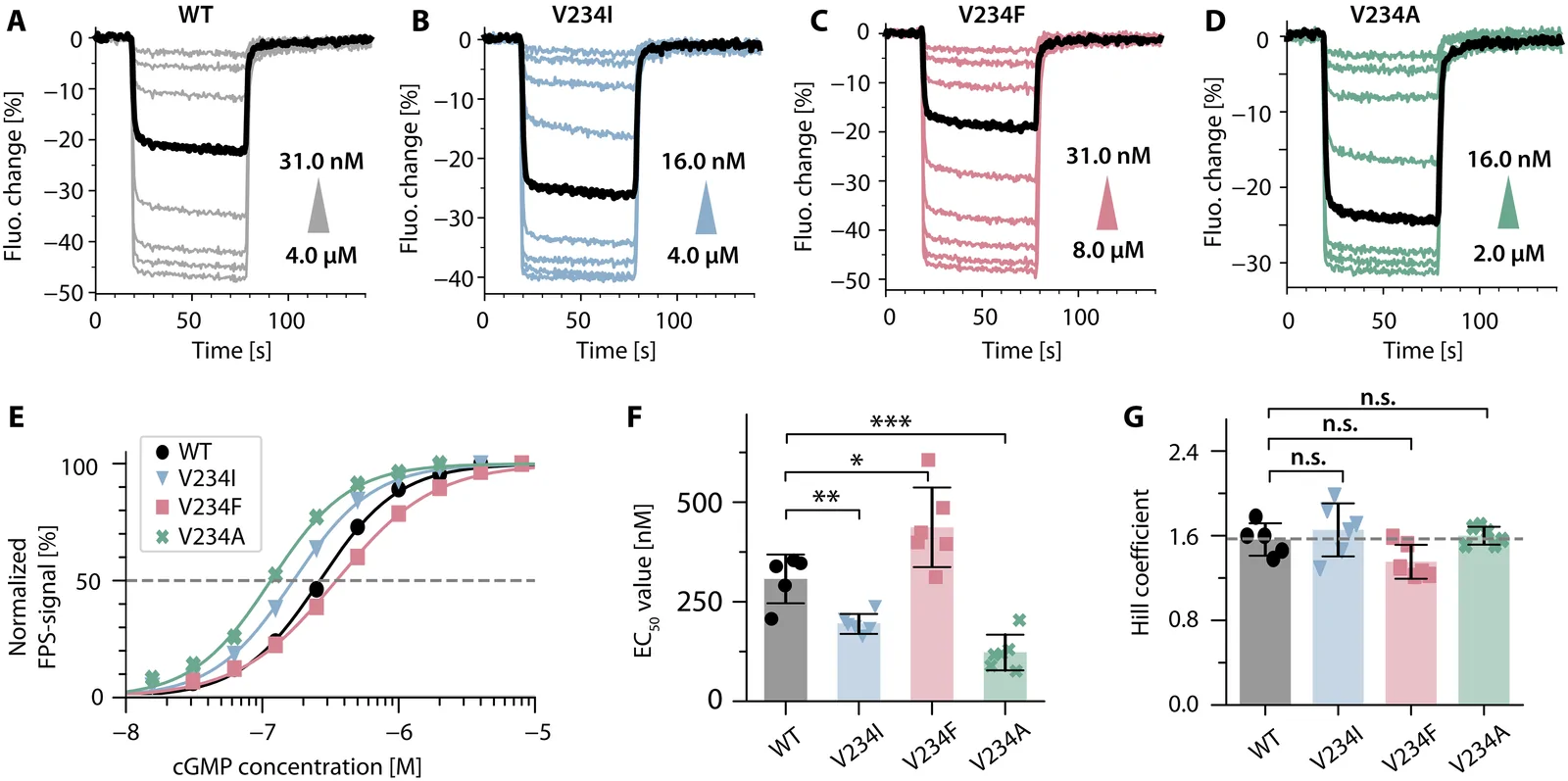
Effect of cGMP on the conformation of PKG Iβ studied with the switchSENSE technology. (**A** to **D**) For switchSENSE measurements, PKG Iβ proteins were captured via conjugated SXT on an Adapter Biochip surface. After 20 s, increasing concentrations of cGMP were applied to the flow channel for 60 s, followed by 60 s of running buffer using a flow rate of 100 μl/min. The relative change in fluorescence is plotted against the measurement time. Fluorescence signals were real-time referenced and average blank subtracted. Response to the 250 nM cGMP sample is highlighted in black. (**E**) The fluorescence change was normalized against the maximal fluorescence change signal detected in the respective experiment. The normalized changes were plotted against cGMP concentration. Data were analyzed using a steady-state analysis with the Hill equation from the heliOS software (version 2025.1.4). (**F**) Quantification of Hill fits with the EC_50_ values. (**G**) Hill coefficients from (E). Data are means ± SD of at least three independent experiments. Significance was tested with an unpaired Student’s *t* test (n.s., nonsignificant, *P < 0.05, **P < 0.01, and ***P < 0.001).

The switchSENSE technology enables the study of conformational changes of proteins in real time by detecting changes in fluorescence (*19*). PKG Iβ WT and the V234I/F/A variants were immobilized, and increasing concentrations of cGMP were introduced in the flow channel. A rapid decrease in fluorescence upon cGMP addition (association), followed by a fast recovery (dissociation), was observed for all variants and WT over a 140-s timeframe (Fig. 3, A to D). These changes result from altered fluorophore quenching caused by cGMP-triggered conformational rearrangements of the immobilized proteins.

Normalized fluorescence changes were plotted against cGMP concentration (Fig. 3E) and fitted using a Hill model to determine median effective concentration (EC_50_) values, corresponding to the cGMP concentration at which 50% of the proteins undergo a conformational transition (Fig. 3F and table S3). The WT exhibited an EC_50_ of ∼300 nM, while the V234I and V234A variants required lower cGMP concentrations to reach the same conformational transition. Unlike V234I and V234A, V234F showed a slightly elevated EC_50_ as apparent from the deviation of V234F from the WT response at higher cGMP concentrations (>300 nM; Fig. 3E). The resulting Hill coefficients for WT and the three V234 variants ranged from 1.36 to 1.66 (Fig. 3G and table S3). Although V234F exhibited a trend toward a Hill coefficient lower than WT and the other variants, these differences were not statistically significant (Fig. 3G). Nevertheless, this trend may be consistent with reduced cooperativity and could contribute to the lower responsiveness of V234F to cGMP.

### V234 variants alter the energetic landscape of cGMP binding in CNB-B

Because the full-length variants displayed distinct cGMP activation profiles as shown by kinase assays (Fig. 2, A to C) and switchSENSE technology (Fig. 3), we directly probed how the V234 substitutions affect cGMP-binding affinities and energetics using ITC on the isolated CNB-B construct. In addition to harboring the V234 variants, the CNB-B domain also serves as the central cGMP-driven control unit of PKG and accounts for its cGMP selectivity (*9*, *20*). The CNB-B is also preserved in both of the PKG I isoforms, Iα and Iβ. The isolated CNB-B of PKG retains cGMP binding affinity comparable to the full-length protein, and its structure remains nearly unaltered upon N- and C-terminal truncation (*8*, *9*, *21*–*23*). Hence, CNB-B constructs are valuable to quantify affinities and map allostery.

**Fig. 4.**
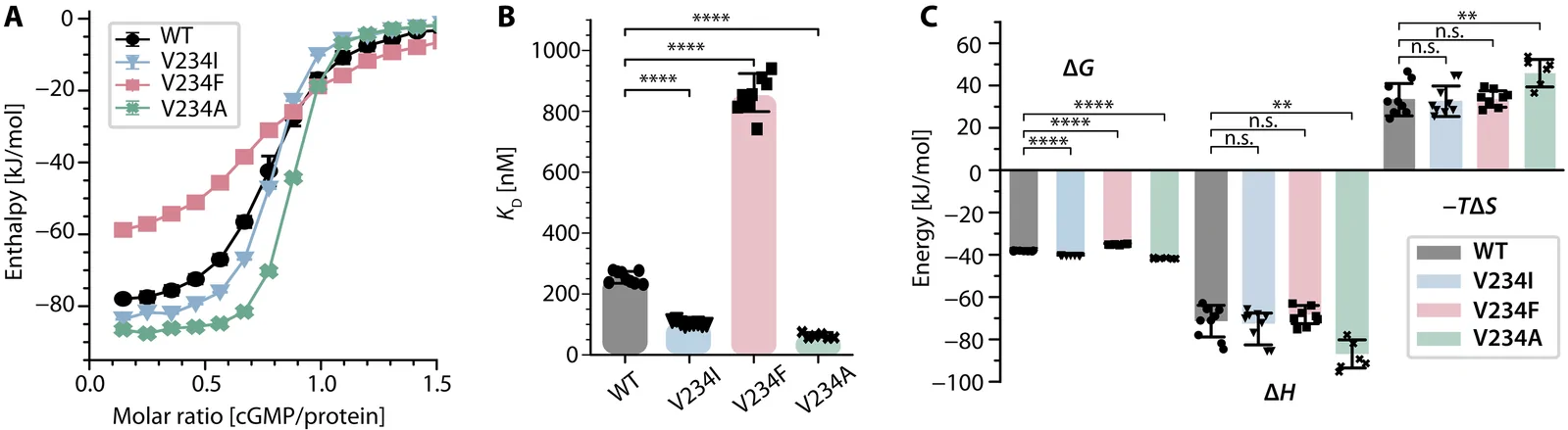
Isothermal titration calorimetry reveals differences in the thermodynamic profiles. (**A**) Thermograms for the binding of cGMP to CNB-B. (**B**) Thermograms were fitted with the independent fit of the NanoAnalyze Software v. 3.12.5 to determine *K*_D_ values for the binding of cGMP. (**C**) Full-thermodynamic profiling with the parameters Δ*G*, Δ*H*, and −*T*Δ*S* were deduced from the independent fit. Data are means ± SD of at least three independent experiments. Significance was tested with an unpaired Student’s *t* test (n.s., nonsignificant, **P < 0.01, ****P < 0.0001).

All V234 variants exhibited exergonic cGMP binding to CNB-B (Fig. 4A), and the interactions were strongly enthalpy driven, similar to WT (Fig. 4C and table S4). WT showed an affinity of ∼250 nM, while V234I and V234A bound more strongly with *K*_D_ values of 100 and 60 nM, respectively (Fig. 4B and fig. S2), in agreement with the kinase assays and switchSENSE profiling, demonstrating that V234I and V234A are more readily activated by cGMP than WT. In contrast, V234F binding affinity to cGMP was ∼3-fold weaker than WT (∼850 nM; Fig. 4B) but was activated at cGMP levels comparable to WT, suggesting that other factors compensate for the loss of cGMP affinity on this variant. To explain the structural origin of these behaviors and probe how CNB-B is structurally reorganized by the different V234 substitutions, we complemented our low-resolution assays (Figs. 2 to 4) with residue-resolution NMR experiments.

### All three V234 variants cause pervasive chemical shift perturbations in the apo CNB-B

**Fig. 5.**
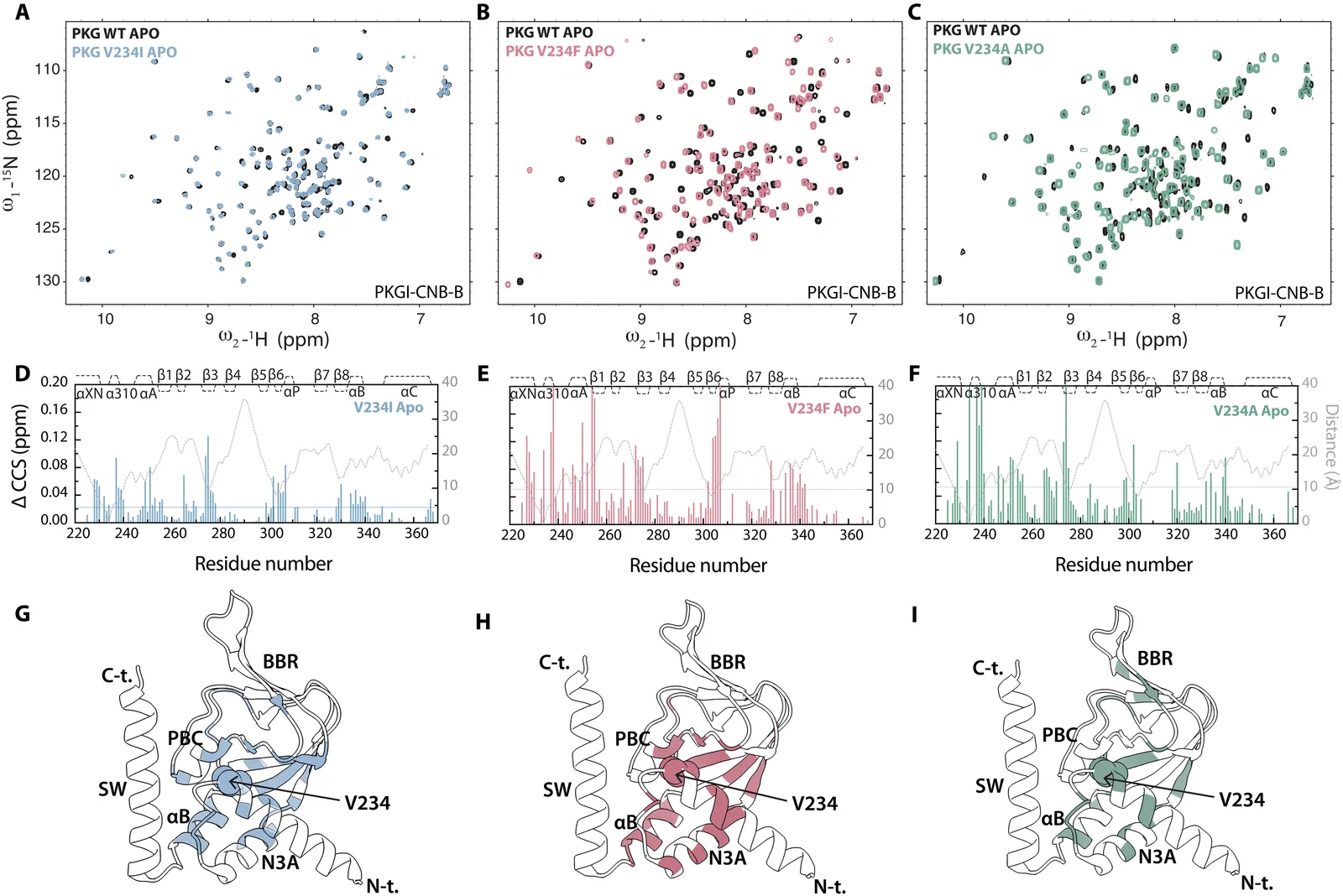
Chemical shift perturbations in the apo PKG I CNB-B upon V234 mutations. (**A**) Overlay of the ^1^H-^15^N-HSQC spectra of WT (black) and V234I (blue), (**B**) V234F (red), and (**C**) V234A (green) in the apo conformation. (**D**) Combined chemical shift (CCS) differences between the WT and the V234I variant for the apo conformation. The gray dashed line plotted on the right (*Y*) axis represents the backbone amide nitrogen distances of each residue from V234 for the apo structure (PDB: 4KU8) in angstrom. The blue dashed line represents the average CCS. Secondary structure features of CNB-B are displayed on top of the graph. (**E** and **F**) Same as (D) but for the V234F and V234A variants, respectively. (**G**) PKG I-CNB-B crystal structure (PDB: 4KU8) highlighting the regions experiencing the most chemical shift perturbations (above average) for V234I (blue). Some key structural elements of domain B are labeled in the structure. (**H** and **I**) Same as (G) but for V234F (red) and V234A (green), respectively.

Comparisons between the ^1^H-^15^N heteronuclear single quantum coherence (HSQC) spectra of PKG I CNB-B V234I/F/A and WT, in the absence of cGMP (apo), revealed pervasive chemical shift changes for all variants (Fig. 5, A to C). In all three variants, the most pronounced chemical shift changes were observed close to the site of the substitution (V234) but also at residues located within a 15-Å radius from V234 (Fig. 5, D to F). The changes produced by V234F were the most drastic compared to the other two mutants, as expected for the bulkier aromatic side chain and possible clash with neighboring residues W304 and I275 (fig. S1F) (Fig. 5E). However, the chemical shift pattern for the three variants was similar, even for V234A (Fig. 5, D to F), which could be explained by the removal of crucial hydrophobic contacts between V234, W304, and I275 (Fig. 5, G to I, and fig. S1, D to G). To gain further insight into the structural changes underlying these chemical shift variations, we next conducted the chemical shift projection analysis (CHESPA).

### All three V234 variants partially shift the apo/inactive conformation of CNB-B toward an active conformation

**Fig. 6.**
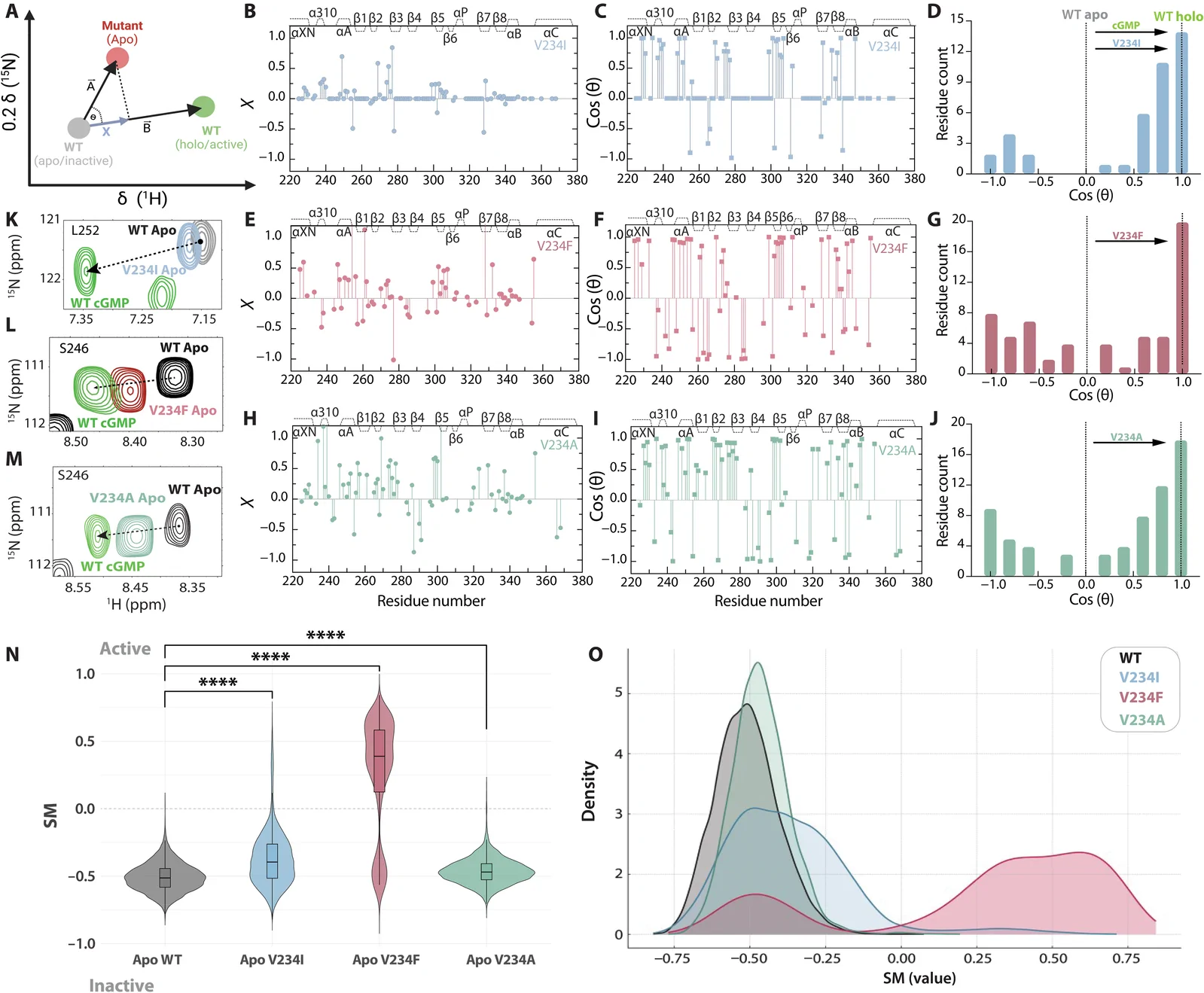
V234 variants shift the inactive CNB-B toward a partially active conformation. (**A**) CHESPA diagram. The WT to mutant chemical shift vector ($\vec{A}$) is projected onto a reference vector ($\vec{B}$) that connects WT inactive/apo to WT active. This projection is used to measure the fractional shift (*X*) and cosine theta [cos (ϴ)]. (**B**) Calculated fractional activation (*X*) values for inactive V234I when projected into a reference vector of WT (inactive) to WT (active). Secondary structure features of CNB-B are displayed above the graph. (**C**) Calculated cos ϴ values for inactive V234I when projected into a reference vector of WT (inactive) to WT (active). Secondary structure features of CNB-B are displayed above the graph. (**D**) Distribution of cos (ϴ) values from (B), displaying a net prevalence of positive cos (ϴ) values. (**E** to **G**) Same as (B) to (D) but for V234F. (**H** to **J**) Same as (B) to (D) but for V234A. (**K** to **M**) Selected ^1^H-^15^N HSQC expansions of residues shifting toward the active conformation upon substitutions at residue 234. (**N**) Violin plots display the distributions of RMSD-based structural MD similarity measures (SMs) calculated for the N3A and β-core regions of PKG CNB-B for each variant and the WT, showing deviation from the apo/inactive starting structure. (**O**) Kernel density plots of SM values for apo WT and PKG I variants (V234I, V234F, and V234A). The *Y* axis represents probability density (1/SM units), scaled such that the area under each curve equals 1. Statistical significance for SM plots was assessed using the Mann-Whitney *U* test (****P < 0.0001).

CHESPA quantifies both the magnitude and direction of the chemical shifts arising from a given allosteric perturbation, in this case, an amino acid substitution (*24*, *25*). Under the assumption of fast exchange (two-state equilibrium), CHESPA can provide information about whether variants shift the equilibrium toward inhibition or activation, with the use of reference ^1^H-^15^N HSQC spectra from the WT in the active (cGMP-bound) and the inactive conformation (apo) (Fig. 6A). For each residue, CHESPA provides the fractional shift (*X*) and the cosine (cos) of the angle (ϴ) between vectors $\vec{A}$ (perturbation vector) and $\vec{B}$ (reference vector) (Fig. 6A). Analysis of the apo spectra of the V234 variants with CHESPA is ideally suited to check whether the substitutions shift CNB-B toward an “active-like” conformation in the absence of cGMP. When the *X* and cos (ϴ) values were computed for V234I, they revealed a net shift toward the active conformation at some key regions, specifically the N3A, as well as select β regions (β3 and β6) and αB (Fig. 6, B and C). This was indicated by most of the residues displaying positive *X* and cos (ϴ) values (Fig. 6, B to D). Similar to V234I, the CHESPA analysis of V234F revealed a shift of the inactive (apo) conformation toward a partially active conformation, as shown by most of the residues having positive *X* and cos (ϴ) (Fig. 6, E to G). However, this time, several residues also showed a shift toward inhibition. Yet, the fractional shift |*X*| of the residues that shifted toward an inactive conformation was less than the |*X*| of residues that shifted toward the active conformation. This is also confirmed by the analysis of the frequency distribution of the cos (ϴ) values (Fig. 6G). Last, V234A also revealed a net shift toward a partially active conformation that is somewhat intermediate between the effects of the V234I and V234F variants (Fig. 6, H to J).

While CHESPA reveals that all three V234 variants bias the apo conformation toward partially active-like states, it does not fully capture the underlying structural dynamics. To further interrogate the structural consequences of these substitutions, we complemented the NMR analysis with MD simulations, tracking deviations from the inactive apo conformation to assess whether the variants “nudge” the domain toward activation.

The conformational landscape of CNB-B was examined using similarity measure (SM) plots derived from >800 ns MD simulations of the apo states. These plots quantify structural deviation [root mean square deviation (RMSD)] relative to the inactive starting structure, with negative values indicating similarity to the inactive state and positive values indicating similarity to the active conformation. The WT apo serves as a reference for the inactive state (mean SM < 0), and an SM value close to 1 or −1 indicates an active- or inactive-like conformation, respectively. SMs of the N3A and β-core regions revealed that the V234 variants sampled conformations with SM distributions shifting toward less negative values (Fig. 6N). V234F, in particular, showed a pronounced shift toward the active state, while V234I showed a less pronounced shift toward the active state. Although minor than V234F and V234I, V234A still exhibits a notable shift toward active conformations compared to WT (Fig. 6N), which is also shown by the distribution of SM densities (Fig. 6O). The MD-derived SMs and the CHESPA analyses both report on shifts in the autoinhibitory conformational equilibrium. The MD simulations show that V234 variants bias the kinase toward more active-like conformations, while CHESPA captures this shift through changes in the NMR cross-peak positions. Together, SMs and CHESPA provide a consistent mechanistic picture in which mutations at V234 promote activation by shifting the conformational equilibrium of PKG–CNB-B toward states with decreased autoinhibitory competency. Overall, our combined chemical shift (CCS) perturbation, CHESPA, and MD analyses strongly point to a central allosteric role of V234. To further investigate the allosteric networks spanning this critical residue, we implemented the chemical shift covariance analysis (CHESCA) (*26*, *27*) of PKG I CNB-B.

### V234 is a core allosteric residue of CNB-B

**Fig. 7.**
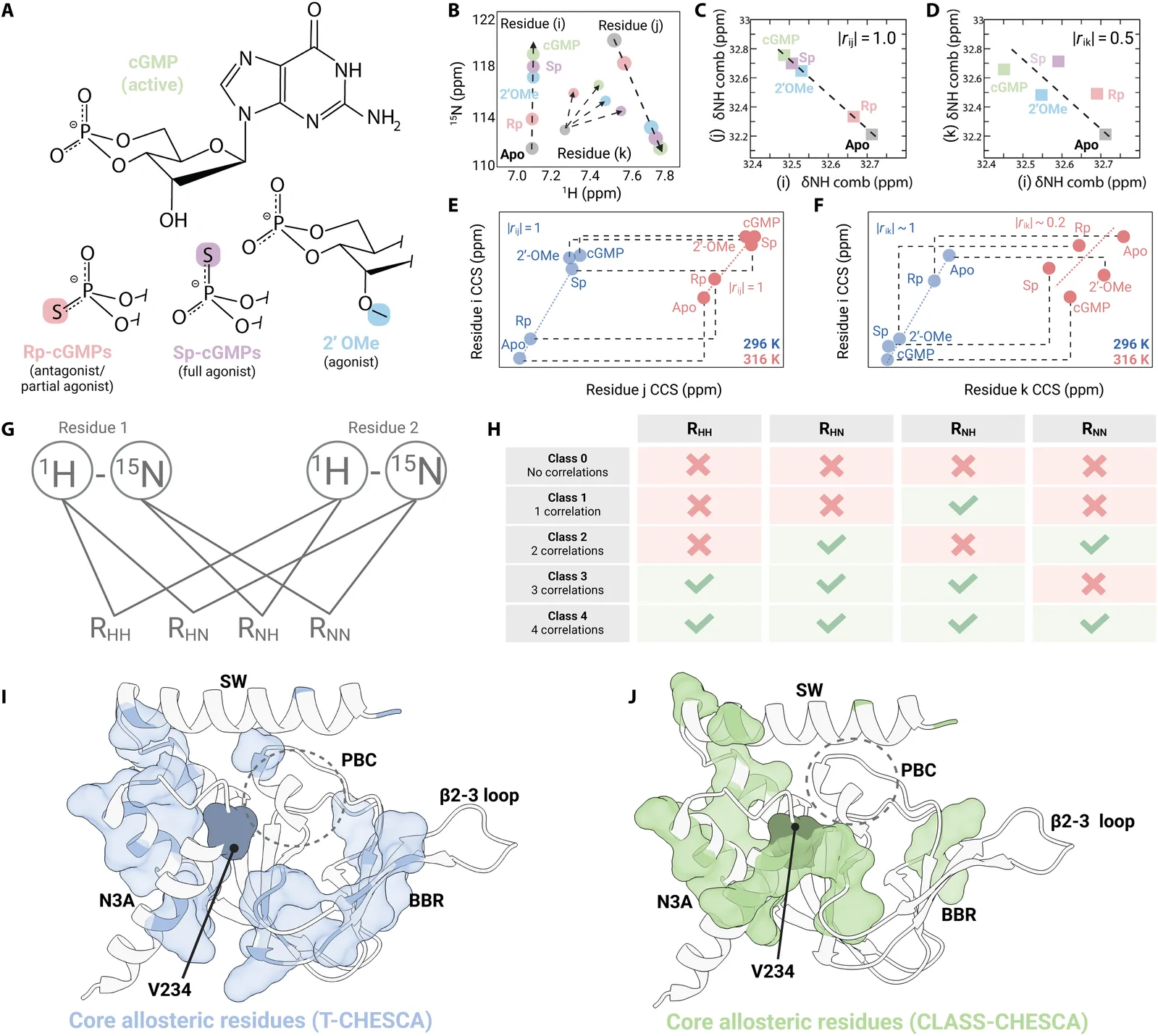
V234 is a core allosteric residue as revealed by T- and CL-CHESCA. (**A**) Perturbation library used for the CHESCA. (**B**) Scheme depicting the chemical shift of three different residues (i, k, and j) upon the binding of different cGMP analogs from the library in (A). Some residues can be subjected to linear chemical shifts (i and j) versus nonlinear chemical shifts (k and i). (**C**) ^1^H and ^15^N CCS correlation plots between two residues (i and j), as presented in (B), experiencing a high degree of correlation when subjected to the same perturbation library. (**D**) Same as (C) but for two residues (i and k) experiencing a lower degree of correlation due to the deviation from linearity of residue k. (**E**) Hypothetical example to explain T-CHESCA rationale: a residue pair (i and j) conserving a high Pearson’s correlation coefficient at low (blue) and high temperature (red). (**F**) Same as (E) but for a residue pair (i and k) that loses their high correlation at a higher temperature (red). (**G**) CL-CHESCA rationale: To capture chemical shift correlations between residues, four Pearson coefficients are computed for each residue pair: R_NH_ (nitrogen shift of the first residue versus proton shift of the second), R_HN_ (proton shift of the first versus nitrogen shift of the second), R_HH_ (proton shifts of both residues), and R_NN_ (nitrogen shifts of both residues). (**H**) In CL-CHESCA, each residue pair is assigned to one of five classes (0 to 4), determined by how many of their four correlation values (R_NH_ to R_NN_) surpass a chosen cutoff (e.g., *R*th = 0.97). (**I**) Core allosteric residues identified by T-CHESCA, including V234, plotted in the crystal structure of PKG–CNB-B (PDB: 4KU8). (**J**) Same as (I) but the core allosteric residues identified by CL-CHESCA.

CHESCA identifies allosteric networks by using correlated NMR chemical shift variations to detect groups of residues that respond similarly to a common perturbation library (Fig. 7, A and B) (*26*). For example, if residues **i** and **j** are allosterically coupled, they display a similar pattern of chemical shift changes upon exposure to a set of perturbations, and, in the limit of fast chemical exchange between inhibitory and noninhibitory conformations, their combined (^1^H and ^15^N) chemical shifts are linearly correlated (|*r*_ij_| ∼ 1) (Fig. 7C). On the other hand, residue pairs not allosterically linked (residues **i** and **k**) give rise to a poor correlation of their CCSs (Fig. 7D).

Although the original CHESCA approach (*26*, *27*) offers an extensive map of allosteric sites, we have developed other related methods, such as temperature-CHESCA (T-CHESCA) and CLASS-CHESCA (CL-CHESCA), aimed at establishing a hierarchy of critical allosteric core residues (*28*). T-CHESCA uses a similar principle to CHESCA, but the CHESCA analysis is performed at different temperatures while still ensuring that the overall folding of the protein is maintained (*28*). Core allosteric residues are defined in T-CHESCA as residue pairs that exhibit a high Pearson correlation coefficient across different temperatures. This is because T-CHESCA assumes that the temperature invariance of pairwise residue correlations is a property unique to residues that are tightly allosterically coupled (Fig. 7E). Residue pairs that do not keep a high correlation coefficient at different temperatures are not considered tightly coupled (Fig. 7F and text S1) (*28*).

When we performed T-CHESCA on PKG I CNB-B at different temperatures (from 296 to 316 K), the residue pairs that kept a Pearson correlation coefficient higher than 0.98 were considered core allosteric residues (Fig. 7I). As expected, an increase in temperature from 296 to 316 K resulted in a substantial loss of correlations in the CNB-B regulatory domain, as seen in the correlation matrices of fig. S3 (A and B). The integrity of the PKG I CNB-B structure was well maintained at high temperatures, as confirmed by the preservation of the chemical shift dispersion (fig. S3C). To select which residue pairs belong to the core allosteric network and maintain high correlations across all temperatures, we computed the mean Pearson coefficient *R* (<*R*>) and its associated ^1^H SD, setting a cutoff of 0.98 (fig. S3D). V234 was identified as one of the core allosteric residues with high correlation coefficients across all different temperatures (Fig. 7I).

Similar to T-CHESCA, CL-CHESCA identifies core allosteric sites with strong couplings. However, instead of temperature, CL-CHESCA varies the type of nuclei used per residue (*28*). As shown in Fig. 7G, CL-CHESCA analyzes ^1^H and ^15^N chemical shifts separately, unlike the original CHESCA method, which combines them into a single CCS per residue (*26*, *28*). While the CCS approach simplifies correlation mapping, it introduces projection compression artifacts where distinct HSQC cross peaks may yield similar CCS values and lead to false positives (text S1). By preserving individual chemical shift dimensions, CL-CHESCA avoids these ambiguities without requiring temperature-dependent measurements. When both ^15^N and ^1^H shifts are available, four interresidue correlation coefficients (*R*_NH_, *R*_HN_, *R*_HH_, and *R*_NN_) are computed to capture all possible nucleus pairings between two residues (Fig. 7G). CL-CHESCA ranks residue pairs into five correlation classes (0 to 4) based on how many of the four nucleus-specific Pearson coefficients (*R*_NH_, *R*_HN_, *R*_HH_, and *R*_NN_) exceed a set threshold (*R*_th_ = 0.97). Strong couplings (classes 3 and 4) are classified as core allosteric sites according to CL-CHESCA (Fig. 7H and fig. S4). Notably, V234 was identified again as a core allosteric residue (Fig. 7J), belonging to class 3. Hence, V234 emerges as one of the core allostery residues of PKG I CNB-B as consistently revealed by both T- and CL-CHESCA (fig. S5).

To further assess to what extent the allosteric networks identified by CHESCA are reflected in the conformational dynamics of PKG CNB-B, we computed the MD-derived dynamic cross-correlation matrix (DCCM). The DCCM analysis reveals correlated motions between the N3A motif, where V234 is located, and distal regulatory elements, including the αB helix, which are also identified by CHESCA as components of the allosteric network (fig. S6, A and B). The overlap of the DCCM and CHESCA matrices supports a shared allosteric communication pathway captured by both approaches (fig. S6C), confirming that V234 is engaged in long-range allosteric couplings.

### V234I and V234A locally destabilize apo CNB-B, while V234F decreases its global stability

**Fig. 8.**
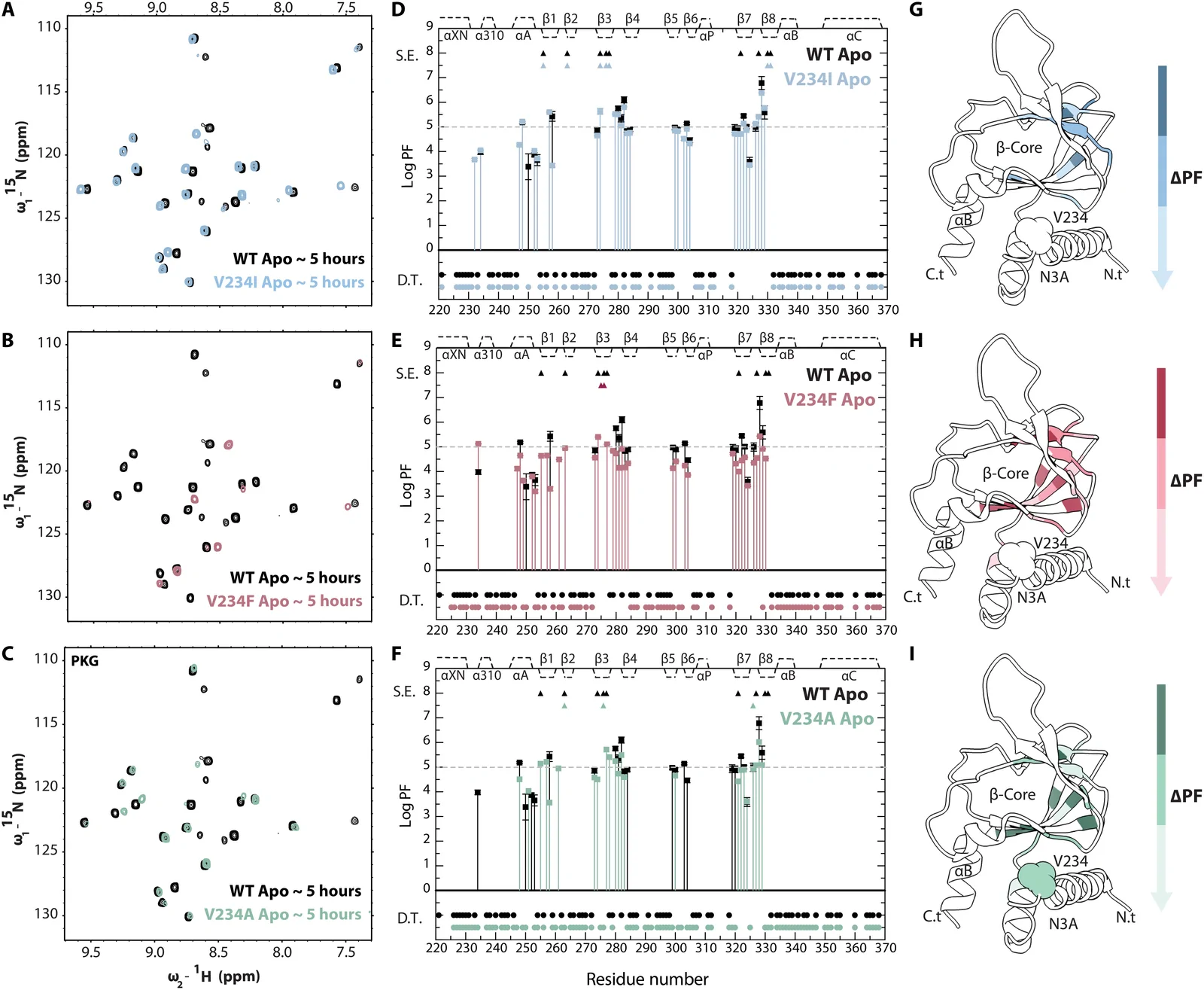
Backbone HD exchange for apo PKG I CNB-B V234 variants. (**A**) Overlay of the ^1^H-^15^N-HSQC spectra of WT (black) and V234I (blue) in the apo conformation 5 hours after exchange from H_2_O to D_2_O. Dead time (∼20 min). (**B** and **C**) Same as (A) but for V234F (B) and V234A (C). (**D**) PFs of the V234I mutant when exposed to D_2_O during HDX NMR. Residues are grouped into three types: residues that exchange within the dead time of the HDX NMR experiment are denoted D.T.; residues, where the hydrogen exchange rate can be quantified, are plotted as the logarithms of PFs that were computed from the HDX NMR data; and residues with very slow exchange rates which were unable to quantify properly are denoted S.E. and are represented by triangles. A gray dashed line is depicted at log PF = 5. (**E** and **F**) Same as (D) but for V234F (E) and V234A (F). (**G**) The residues whose PF was measured in the HDX experiment of V234I were plotted in the crystal structure of PKG–CNB-B (PDB: 4KU8). The color code identifies residues experiencing the most PF change (darker shade) to the least (lighter shade). (**H** and **I**) Same as (G) but for V234F (H) and V234A (I).

Having identified V234 as part of the core allosteric network, we next asked whether its substitution induces changes in CNB-B’s local and global folding stability by measuring hydrogen-deuterium exchange (HDX) rates by NMR. A simple qualitative comparison of the apo WT and apo V234I spectra after 5 hours in the D_2_O exchange buffer showed a similar pattern of solvent protection (Fig. 8A). However, calculation of the protection factors (PFs), which quantitatively reflect how much a backbone amide proton is shielded from the solvent, revealed specific residues in the V234I variant that are more exposed compared to the WT (Fig. 8D). One clear example is residue E258, with log PF of ∼5.5 in WT but ∼3.5 in V234I, pointing to two order of magnitude reduction for this PF (Fig. 8D and fig. S7). Although selected residues are more exposed, the replacement of Val for Ile does not have a drastic effect on the global folding stability of the domain, as shown by the marginal changes observed for the highest protection of factors of the inner β barrel strands of CNB-B (β3, 4, 7, and 8; Fig. 8D), which are known to report on global unfolding (*20*, *29*).

When we carried out the same HDX experiment for apo V234F, the results were notably different. A comparison of the ^1^H-^15^N HSQC spectra of the variant with WT revealed substantial changes after the exchange occurred for ∼5 hours (Fig. 8B). Most of the signal of V234F was lost. This observation already points out that V234F causes a drastic exposure of residues that are buried in WT. When residue-specific log PF values were computed, we observed an overall notable loss for most residues in the β-subdomain (Fig. 8E). In addition, several slowly exchanging residues previously detected for WT were no longer in the slow HDX time scale (Fig. 8E, upward triangles). The analysis of the HDX of apo V234A revealed an intermediate pattern between V234I and V234F. The loss of β-subdomain protection observed for the V234A variant is larger than for V234I but smaller than for V234F (Fig. 8, C, F, and I), and compared to V234F, a higher number of slowly exchanging residues are observed for V234A after 5 hours of exposure (Fig. 8, B and C).

To quantify the relative hierarchies of folding stabilities for the variants, we converted residue-specific PF values into opening free energies (Δ*G*_op_), following the pioneering HDX analyses by Bai *et al.* (*30*–*34*) and assuming that at our neutral pH, an EX2 Linderstrøm-Lang exchange mechanism, applies, resulting in Δ*G*_op_ = RTln(PF). To enable direct comparisons of stability changes across variants, we focused on a common set of highly protected residues shared across WT (log PF ≥ 5) and all variants (i.e., residues 248, 258, 280 to 282, 303, 322 to 323, and 328 to 329, which include the β barrel inner β strands 3, 4, 7, and 8). Using this common stable core of the CNB-B domain, we observe a progressive destabilization of the CNB-B domain, with mean Δ*G*_op_ values of 7.2 ± 0.2 kcal/mol for WT, 6.9 ± 0.2 kcal/mol for V234I, 6.5 ± 0.3 kcal/mol for V234A, and 6.1 ± 0.2 kcal/mol for V234F. This trend is consistent with a mutation-dependent decrease in global stability, which is most pronounced for V234F. While V234F induces broad destabilization across the domain, V234I shows more localized perturbations. V234A shows intermediate effects. All variants remain folded, indicating destabilization rather than global unfolding. In addition, in agreement with the CHESCA results (Fig. 7, I and J, and fig. S6B), comparison of the three-dimensional (3D) maps for HDX and CCS (fig. S8) illustrates how mutations at V234 cause perturbations that cross the α/β-subdomain interface to reach the β barrel, where most of HDX-detectable changes are confined.

## DISCUSSION

**Fig. 9.**
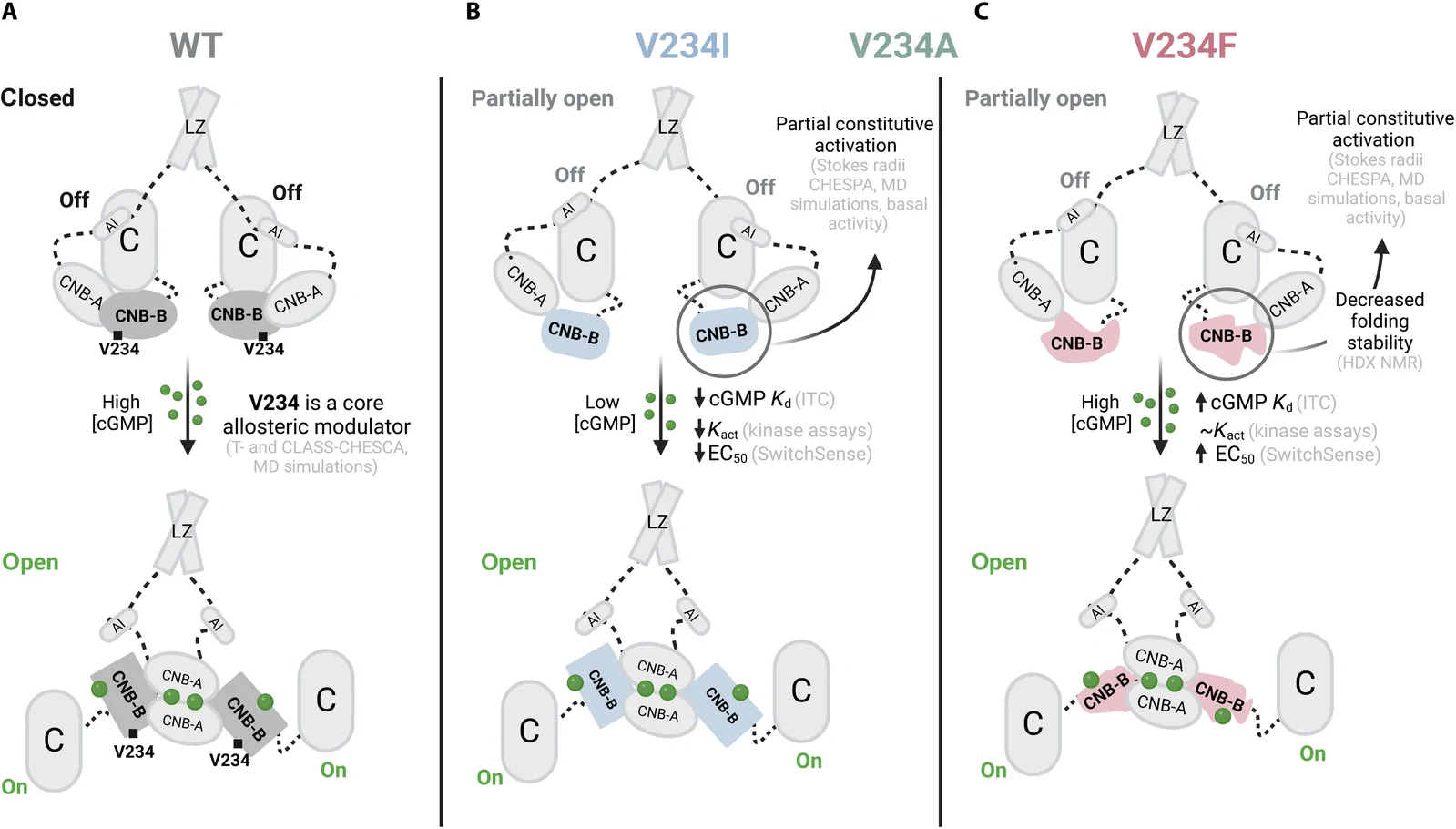
Schematic mechanism of action of the V234 disease-associated variants in PKG Iβ. (**A**) WT transitions from the inactive (closed) conformation to the active (open) conformation. (**B**) Same as (A) but for partially open V234I and (**C**) for V234F. Since the V234A mechanism of action resembles V234I, besides enhanced local destabilization, V234A is depicted closer to (B) than (C). Evidence supporting specific features of the mechanisms is depicted in light gray text. Created in BioRender. Martinez Pomier, K. (2026) https://BioRender.com/6j16scc.

We have identified the highly conserved V234 (fig. S1) as one of the core allosteric hubs of PKG I CNB-B, as revealed by both T- and CL-CHESCA. The comparative functional and biophysical analyses of three variants at this site, i.e., V234I, V234A, and V234F, support two distinct mechanisms that explain the PKG overactivation underlying TAAD caused by these PKG mutants (Fig. 9, A to C). The two mechanisms are epitomized by V234I and V234F (Fig. 9, B and C) and share the common feature of partial constitutive kinase activation. This occurs without altering the maximal catalytic activity at saturating cGMP levels and results from a partial shift toward active conformers in the apo CNB-B inactive-active equilibrium, as consistently supported by multiple independent lines of evidence. These include CHESPA, MD simulations, and increased SEC-based Stokes’ radii, which have been associated with more open PKG topologies and PKG activation in previous reports (*35*, *36*). However, in the case of V234F, the increased basal activity also reflects additional contributions from enhanced global destabilization of apo CNB-B, as shown by HDX. The reduced folding stability of V234F CNB-B compromises autoinhibitory contacts formed between the kinase domain and CNB-B in the absence of cGMP (*8*) and defines a net differential between the two mechanisms embodied by V234I and V234F (Fig. 9, B and C).

Another critical difference between the mechanisms exemplified by V234I and V234F pertains to the cGMP dependence of PKG I activation (Fig. 9, B and C). In the case of V234I, kinase assays and switchSENSE data clearly indicate sensitization to cGMP. ITC experiments show that this is rationalized by increased cGMP affinity of V234I versus WT (Fig. 9B). The increase in the affinity of V234I for cGMP in turn is explained by the larger population of active CNB-B conformers in V234I versus WT, as active states bind cGMP more tightly than inactive states. Unlike V234I, in the case of V234F, no sensitization to cGMP was detected by kinase assays, while switchSENSE data point to increased EC_50_ values, which reflect a decreased cGMP affinity of V234F versus WT CNB-B, as revealed also in the *K*_D_ values for cGMP determined by ITC (Fig. 9C). The decrease in the affinity of V234F for cGMP is accounted for by the global destabilization of CNB-B caused by this variant, as shown by HDX NMR. As the enhanced destabilization of V234F versus WT weakens the inhibitory contacts of CNB-B with the kinase domain (Fig. 1B), V234F exhibits a *K*_act_ value similar to WT despite ∼3-fold weaker cGMP binding (∼800 nM). Last, the V234A mutant displays a mixed pattern, indicating that both mechanisms (Fig. 9, B and C) are, to some extent, concurrently at play. Like V234I, V234A exhibits sensitization to cGMP, yet it is also subject to partial destabilization, as with V234F. The V234A CNB-B fold is more stable than V234F but less stable than V234I, consistent with the loss of hydrophobic packing interactions with W304 and I275 (fig. S1) as previously suggested by the chemical shift perturbation data.

In conclusion, our study explains at the structural and functional level the pathogenicity of aortopathy-related PKG I variants targeting the allosteric “hot spot” residue V234. Although V234 is distal from both the cGMP binding site and the catalytic domain, variations of this core allosteric residue lead to kinase hyperactivity and disease, such as TAAD. Two primary underlying mechanistic routes emerge: Variants such as V234I bias CNB-B toward a stable, partially active-like conformation that enhances cGMP sensitivity, whereas variants such as V234F destabilize the regulatory fold itself, weakening autoinhibitory contacts. Both mechanisms converge on the same outcome: loss of regulatory restraint and elevated PKG activity, observed at both basal and intermediate but not maximal cGMP levels. These two mechanisms are not mutually exclusive and can occur simultaneously, as in the case of the V234A variant, providing a general structural rationale for how V234 substitutions, and possibly other variants, drive pathogenic signaling, and affect disease progression.

## MATERIALS AND METHODS

### Site-directed mutagenesis

#### Isolated cyclic nucleotide binding domain B (CNB-B)

The V234 variants were engineered through site-directed mutagenesis using a pQTEV plasmid containing the WT CNB-B of PKG I (219 to 369 in the Iβ nomenclature) as a template. The DNA was verified through Sanger sequencing of the resulting plasmids. The primers used are as follows: V234I: TTTTTAAAAAGCATCCCAACATTCCAGAGC (forward) and GCTCTGGAATGTTGGGATGCTTTTTAAAAA (reverse); V234F: TTTTTAAAAAGCTTCCCAACATTCCAG AGC (forward) and GCTCTGGAATGT TGGGAAGCTTTTTAAAAA (reverse); V234A: TTTTTAAAAAGCGCTCCAACATTCCAGAGC (forward) and GCTCTGGAATGTTGGAGCGCTTTTTAAAAA (reverse).

#### Full-length protein

The full-length protein constructs were generated by site-directed mutagenesis with the primers listed as follows using a pcDNA3.0 vector with the PKG Iβ sequence (amino acids 5 to 686) and verified by Sanger sequencing. V234I: GGAATTTTTAAAAAGCATTCCAACATTCCAGAG (forward) and CTCTGGAATGTTGGAATGCTTTTTAAAAATTCC (reverse); V234F: GGAATTTTTAAAAAGCTTTCCAACATTCCAGAG (forward) and CTCTGGAATGTTGGAATGCTTTTTAAAAATTCC (reverse); V234A: GGAATTTTTAAAAAGCGCTCCAACATTCCAGAG (forward) and GGAATTTTTAAAAAGCGCTCCAACATTCCAGAG (reverse).

### Protein expression and purification

#### CNB-B for NMR experiments

As previously described (*20*, *37*), a pQTEV plasmid containing domain B of PKG I (219 to 369 in the Iβ nomenclature) with an N-terminal poly-l-histidine for the WT as well as the V234 variants was expressed in *Escherichia coli* strain BL21(DE3). The bacterial cells were cultured at 37°C with ampicillin (50 μg/ml) in ^15^N-ammonium chloride–enriched M9 minimal media containing trace metals, d-biotin, and thiamine-HCl. Protein expression was induced by adding 0.5 mM isopropyl-β-d-thiogalactopyranoside (IPTG) when the optical density reached ∼0.8 OD_600_. The cells were then incubated for 18 hours at 20°C before being collected via centrifugation at 10,000*g* for 10 min and stored at −80°C until purification.

For purification, the cell pellets were initially resuspended in lysis buffer [50 mM tris-HCl (pH 7.6), 150 mM NaCl, 1 mM β-mercaptoethanol, and 0.2 mM 4-(2-aminoethyl) benzenesulfonyl fluoride protease inhibitor]. This step was then followed by lysing the cells in a cell disruptor (Constant Systems, United Kingdom) at 20 PSI (139 kPa). The lysed cells were then removed by centrifugation at 13,000 rpm for 1 hour. Next, the CNB-B domain of PKG1 was isolated using a Ni^2+^-Sepharose resin packed in a 5-ml gravity column. The column was first washed with the lysis buffer, followed by a second wash with the wash buffer [50 mM tris-HCl (pH 7.6), 500 mM NaCl, 20 mM imidazole, and 1 mM β-mercaptoethanol]. Last, the protein was eluted using elution buffer [50 mM tris-HCl (pH 7.6), 50 mM NaCl, 300 mM imidazole, and 1 mM β-mercaptoethanol]. The elution was then dialyzed overnight in cleavage buffer [50 mM tris-HCl (pH 7.6), 100 mM NaCl, and 1 mM β-mercaptoethanol] to remove the excess of imidazole. After dialysis, the eluant was combined with tobacco etch virus (TEV) protease for 48 hours to cleave the poly-l-histidine tag from the PKG I CNB-B (219 to 369) construct.

A second Ni^2+^-Sepharose gravity column was used to remove the cleaved poly-l-histidine tag and TEV. The protein was further purified using size exclusion fast protein liquid chromatography on a HiLoad 16/60 Superdex 200 increase gel filtration column (GE Healthcare). The column was first equilibrated with SEC or NMR buffer [50 mM tris-HCl (pH 7.0), 100 mM NaCl, 1 mM dithiothreitol, and 0.02% (w/v) NaN_3_]. The NMR buffer was used for all the NMR experiments with the exception of HDX NMR experiments, which were performed in a D_2_O-based phosphate buffer [20 mM NaH_2_PO_4_/Na_2_HPO_4_ and 50 mM NaCl (pH 7.0)].

#### CNB-B for ITC measurements

PKG CNB-B (219 to 369 in Iβ nomenclature) in a pQTEV vector was expressed in *E. coli* TP2000 cells, grown to OD_600_ ∼ 1.0 at 37°C, and induced with 400 μM IPTG. Induced cells were cultivated overnight at room temperature, harvested by centrifugation, and stored at −20°C until purification.

Cell pellets were thawed on ice and resuspended in lysis buffer [50 mM NaH_2_PO_4_/Na_2_HPO_4,_ 300 mM NaCl, 10 mM imidazole, 0.5 mM tris(2-carboxyethyl)phosphine (TCEP), and EDTA-free protease inhibitor (Roche, Germany) (pH 8.0)]. Cells were ruptured via FRENCH Pressure Cell Press (Thermo Fisher Scientific, Waltham, USA) with a pressure of 1000 psi (6894.76 kPa). After centrifugation (20 min, 40,000*g*), the clear supernatant was incubated with Ni-NTA agarose (Machery-Nagel, Düren, Germany) on a rotating wheel for 1 hour. The agarose slurry was transferred into a Pierce column equipped with filters. The column was washed three times with wash buffer [50 mM NaH_2_PO_4_/Na_2_HPO_4_, 300 mM NaCl, and 25 mM imidazole (pH 8.0)]. Elution was performed with elution buffer [50 mM NaH_2_PO_4_/Na_2_HPO_4_, 300 mM NaCl, and 100 mM imidazole (pH 8.0)]. Protein constructs were dialyzed against storage buffer [10 mM Hepes and 150 mM NaCl (pH 7.3)].

#### Full-length proteins

Human embryonic kidney 293T cells were grown in whole medium (Dulbecco’s modified Eagle’s medium, 10% fetal calf serum, and 1% penicillin/streptomycin, Capricorn Scientific; Ebsdorfergrund, Germany) with final concentrations of penicillin (100,000 U/liter) and streptomycin (100 mg/liter), to a confluency of 80% before transfection with the pcDNA3.0 plasmid carrying the sequence for the FLAG-Strep-Strep (FSS)–PKG Iβ protein (WT and variants) with polyethyleneimine (Polysciences Europe GmbH, Hirschberg an der Bergstraße, Germany) as previously described (*8*). Cells were harvested by centrifugation and kept frozen at −20°C until usage.

Cell pellets were thawed and homogenized in lysis buffer [20 mM Hepes, 150 mM NaCl, 0.5 mM TCEP, protease and phosphatase inhibitors (Roche, 1 tablet per 40 ml), and 0.4% P20 (pH 7.3)]. After centrifugation (20 min, 40,000*g*, 4°C), the protein-containing supernatant was applied to Streptactin Superflow resin (IBA Lifesciences Goettingen, Germany) and washed with lysis buffer [four column volumes (CVs)]. Next, a wash step with a phosphate buffer [366 mM Na_2_HPO_4_, 134 mM NaH_2_PO_4_, and 0.5 mM TCEP (pH 7.3); four CVs] was conducted at room temperature, followed by two additional wash steps with storage buffer [20 mM Hepes, 150 mM NaCl, and 0.5 mM TCEP (pH 7.3); four CVs] at 4°C. The protein was eluted with elution buffer [200 mM tris-HCl, 300 mM NaCl, 2 mM EDTA, and 5 mM desthiobiotin (pH 8.0)], followed by a buffer exchange into storage buffer as previously described (*8*).

### Kinase activity and activation assays

Kinase activity was determined with a coupled spectrophotometric assay, as previously described (*38*) using VASPtide (RRKVS*KQE, 1 mM) as substrate (*18*). The reaction was followed by the decrease in absorbance at 340 nm using a plate reader (Clariostar, BMG Labtech, Ortberg, Germany). For the determination of activation constants serial dilutions of cGMP (25 μl) were added to 384-well plates (clear bottom, Brand, Germany) and incubated with 25 μl of 20 nM protein solution (final PKG I concentration in the assay: 5 nM). The reaction was started by the addition of 50 μl of Cook Assay Mix containing 100 mM MOPS, 10 mM MgCl_2_, 1 mM adenosine triphosphate, 1 mM phosphoenolpyruvate, lactate dehydrogenase (15.1 U/ml), pyruvate kinase (8.4 U/ml), ∼230 μM NADH (reduced form of nicotinamide adenine dinucleotide), and 0.5 mM TCEP. Activation constants were determined using a sigmoidal dose response plot (GraphPad Prism 8). A total of 8 μM cGMP was used to determine the maximal kinase activity, while samples without cGMP reflect the basal activities of PKG.

### Size exclusion chromatography

Running buffer [10 mM Hepes, 150 mM NaCl, 50 μM EDTA, and 50 μM TCEP (pH 7.3)] was degassed before measurements. A Superose-6-Increase 3.2/300 column (GE Life Sciences, Watman, USA) was equilibrated with two CVs of running buffer. Forty microliters of protein sample with a final concentration of 4 μM, pretreated by centrifugation (10 min, 40,000*g*, 4°C), was loaded onto the column using a 25-μl sample loop. Experiments were conducted at 4°C with flow rates between 40 and 60 μl/min. Samples were automatically injected after equilibration of the column with 0.3 ml of running buffer in each run. Absorbance was continuously recorded at 280 nm, and peak retention times were detected. The column was calibrated using a commercial standard (catalog no. 1511901, Bio-Rad, Munich, Germany) containing the proteins thyroglobulin, bovine gamma-globulin, chicken ovalbumin, equine myoglobin, and vitamin B12 with known Stokes’ radii (*R*_s_). The distribution coefficient (*K*_Av_) of the respective protein was plotted against the log_10_ of *R*_s_. Stokes’ radii were calculated by applying the following formula

$$R_{s}=10^{\frac{K_{\text{Av}}-b}{m}}$$

with m as the slope of the linear regression and b as the *y* intercept of the linear regression (*39*, *40*).

### SwitchSENSE technology

Measurements were conducted on a heliX Adapter Biochip in a heliX+ device. DNA ligand strands were conjugated with Strep-Tactin XT (SXT conjugates; IBA Lifesciences, Goettingen, Germany) via primary amines (amine coupling kit, Bruker Biosensors, Munich, Germany). These conjugates were purified using the proFIRE platform (Bruker Biosensors, Munich, Germany), and the concentrations were determined via spectrophotometric measurements at an absorbance of 260 nm according to the manufacturer’s manual. SXT conjugates were diluted in running buffer [10 mM Hepes, 150 mM NaCl, 50 μM EDTA, 50 μM TCEP, and 0.05% P20 (pH 7.3)] to 500 nM and hybridized with Adapter strand 1-Ra (Bruker Biosensors, Munich, Germany) for 20 min at 25°C, under constant shaking (600 rpm) before adding them to an Adapter strand 2-Ra ligand free strand (Bruker Biosensors, Munich, Germany) in a 1:1 ratio. FSS-tagged proteins were diluted to 50 nM in running buffer with bovine serum albumin (BSA; 0.1 mg/ml) before the capture step (flow = 50 μl/min, 120 s).

After a short baseline with a running buffer (20 s), increasing concentrations of cGMP, diluted in running buffer, were applied to the chip surface for 60 s to determine the association, followed by 60 s of dissociation with running buffer both at a flow rate of 100 μl/min. Fluorescence signals were constantly recorded. Signals were real-time referenced and blank-subtracted using the heliOS software. Data evaluation was done via steady-state analysis, where data between 20 and 50 s of the association phase were averaged and plotted against the used cGMP concentration and evaluated using the Hill Fit of the heliOS software (see text S2) (version 2025.1.4). Visualization was performed with matplotlib (*41*).

### Isothermal titration calorimetry

Samples were measured at the ITC devices (Affinity ITC SV, Affinity ITC) LVTA Instruments, New Castle, USA) with different cell volumes [1 ml (SV) and 200 μl (LV)]. PKG I CNB-B was dialyzed against ITC experimental buffer [10 mM Hepes and 150 mM NaCl (pH 7.3)] three times in 1 liter each. After dialysis, protein concentrations were determined via Bradford assay using a BSA standard curve. Protein samples were diluted to 10 μM (SV device) and 40 μM (LV device) and placed in the sample cell. cGMP was diluted in experimental buffer (400 μM) and placed in the injection syringe before being titrated into the respective sample cell. Titration curves are shown in fig. S2.

### Statistical analysis

For kinase assays, switchSENSE, and ITC experiments, the data distribution was tested using the Kolmogorov-Smirnov test. Then, an unpaired Student’s *t* test was applied (GraphPad Prism v.8.0.1) to determine statistical significance between WT and the V234 variants. For MD simulations, SM statistical significance was assessed using the Mann-Whitney *U* test, with differences in overall distribution shape evaluated using the Kolmogorov-Smirnov test in GraphPad Prism v.10.

### NMR experiments and data analysis

All NMR spectra were acquired in a Bruker AV 700 spectrometer equipped with a TCI cryoprobe at 306 K. For the chemical shift–based experiments such as ∆CCS and CHESPA of PKG I CNB-B, 2D ^1^H-^15^N HSQC spectra were acquired for unbound-inactive (apo) and bound-active (cGMP) conformations, with a protein concentration of 50 μM and 1 mM of cGMP. In addition, 80 μM ^15^N-labeled *N*-acetylglycine was added to the NMR samples as an internal reference. The acquisition parameters of the HSQC experiments were a recycle delay of 1.0 s, 64 scans, and 2 K (t_2_) and 198 (t_1_) complex points for spectral widths of 16.23 parts per million (ppm) (^1^H) and 38.00 ppm (^15^N). The spectra were processed with Bruker TopSpin 4.0.7 and analyzed with Poky (*42*, *43*). The peak assignments for WT were previously resourced from standard 3D triple-resonance NMR experiments, as previously described (*20*). For the ^1^H-^15^N CCS differences (ΔCCS) analysis, the apo spectra of PKG I CNB-B WT, as well as the apo variants (V234I/F/A), were aligned in Poky (*43*), and ΔCCS were computed as described previously (*20*, *44*), using the following equation

$$\text{ΔCCS}=\sqrt{\left\{{\left[\delta_{H,1}-\delta_{H,2}\right]}^{2}+{\left[0.2\left(\delta_{N,1}-\delta_{N,2}\right)\right]}^{2}\right\}}$$

where δ are the proton (H) and nitrogen (N) chemical shifts in parts per million for a given state (i) and a reference (ii). In addition, the resulting ^1^H and ^15^N chemical shifts were used to perform the CHESPA, as described previously (*25*, *27*).

To further analyze the PKG I CNB-B stability, HDX NMR experiments were conducted for the WT and variants as previously described (*20*, *45*). Briefly, amide proton HDX rates were determined from time-dependent changes in ^1^H-^15^N HSQC cross-peak intensities. Apo PKG I CNB-B WT and V234 variants were exchanged into 100% D_2_O buffer [20 mM phosphate buffer and 50 mM NaCl (pH 7.0)] with a PD-10 desalting column Sephadex G-25 resin (Cytiva, Canada), and a series of ^1^H-^15^N HSQC spectra (86 per condition) were acquired as a function of time. The time between sample exchange into D_2_O and data acquisition (dead time) was estimated to be ∼20 min. HDX was sampled over the first 24 hours following exchange into D_2_O. For slowly exchanging residues, an additional late time point (∼1 week) was acquired to assess long-lived protection. This strategy enables quantification of intermediate exchange rates while allowing identification of highly protected residues. The NMR data were processed and analyzed with NMRPipe (*46*), and Curvefit with the Levenberg-Marquardt nonlinear least square exponential fitting was used to determine the exchange rates per residue. PFs were obtained by comparing experimentally measured exchange rates with intrinsic rates predicted by SPHERE for a polyalanine reference (*33*), as previously described (*29*, *45*). PFs were computed for residues experiencing quantifiable solvent exchange. Residues that exchanged within the dead time of the experiment were considered fast exchangers, while residues for which the exchange was too slow for reliable PF quantification were considered HDX slow exchangers.

### MD simulations

#### Model preparation

Initial structural models for the MD simulations were constructed from available x-ray crystal structures of PKG Iβ CNB-B (residues 217 to 351). An apo/inactive state structure for WT CNB-B was derived from the x-ray structure of cGMP-free CNB-B [Protein Data Bank (PDB) ID: 4KU8] by deleting all water molecules, extra ligands (present after protein crystallization), and extra copies of the protein from the x-ray structure, using SwissPDB Viewer (*47*) to reconstruct partially missing side chains on the protein surface. Similarly, a cGMP-bound/active state structure for WT CNB-B was derived from the x-ray structure of cGMP-bound CNB-B (PDB ID: 4KU7), using the online “ModLoop” module of Modeller (*48*) to reconstruct a missing loop in the x-ray structure (residues 287 to 291). Initial simulation structures with hydrogen atoms, and corresponding molecular structure topology files, were then generated from the constructed structural models via the “psfgen” module of VMD 1.9.3 using the CHARMM27 all-atom force field, following a protocol similar to that described previously (*49*). Corresponding initial structures for the disease-associated variants of CNB-B (i.e., V234I, V234F, and V234A) were achieved by introducing each substitution into the WT structures, using the intrinsic “mutate” tool of the psfgen module of VMD 1.9.3, during generation of the initial structures and topology files.

#### MD simulation protocol

MD simulations were performed starting from the apo/inactive WT and V234I/F/A mutant structures of PKG I CNB-B using the NAMD 2.12 software (*50*) on the Shared Hierarchical Academic Research Computing Network. All simulations used the CHARMM27 force field with CMAP correction, and simulation setup and execution were performed following a protocol similar to that described previously (*51*), with solvent box dimensions of 86 Å. All simulations were executed using 2.1-GHz 32-core Broadwell compute nodes accelerated with two NVIDIA Pascal GPUs per node. The simulations were each executed for >800 ns at constant temperature and pressure, saving structures every 100,000 time steps (i.e., 100.0 ps) for subsequent analysis.

#### Structure SM

To assess the propensity of apo/inactive WT and V234I/F/A CNB-B structures to sample active- or inactive-like conformations, the MD trajectories were analyzed by computing RMSD-based active-versus-inactive structural SMs, following a protocol similar to that described previously (*49*). Backbone RMSDs from the inactive and active CNB-B conformations were computed for the N3A/β-core region (overlaying residues 224 to 251, 253 to 264, 271 to 285, 294 to 305, and 319 to 333, excluding flexible loops which could affect the structural alignment during the RMSD calculations) of WT and each variant over the course of the respective MD trajectories, using the corresponding inactive and active structural models as reference structures for the calculations. The corresponding SM values were then calculated from these RMSDs as described previously (*49*).

#### DCCM analysis

Correlated motions in the MD trajectories were analyzed using DCCM calculations performed in cpptraj (*52*) (AmberTools) (*53*). Before the analysis, each trajectory was aligned to the first frame using a least-squares fit on the Cα atoms to remove overall rotational and translational motions. The DCCM was then computed using the fluctuations of Cα atomic coordinates over the trajectory. The correlation coefficient between residues *i* and *j* was calculated as

$$C_{\mathrm{ij}}=\frac{\left\langle \left(r_{i}-\left\langle r_{i}\right\rangle \right)\cdot \left(r_{j}-\left\langle r_{j}\right\rangle \right)\right\rangle }{\sqrt{\left\langle {\left(r_{i}-\left\langle r_{i}\right\rangle \right)}^{2}\right\rangle \cdot \left\langle {\left(r_{j}-\left\langle r_{j}\right\rangle \right)}^{2}\right\rangle }}$$

where $r_{i}$ and $r_{j}$ are the position vectors of the Cα atoms of residues *i* and *j*, respectively, and angle brackets denote averages over the trajectory. The resulting correlation coefficients range from +1 (fully correlated motion) to −1 (fully anticorrelated motion), with 0 indicating no correlation. The correlation matrices were exported from cpptraj and visualized in Python using matplotlib (*41*) as heatmaps.

### T- and CL-CHESCA

For the T- and CL-CHESCA experiments, ^15^N-^1^H HSQC was acquired as previously described (*28*) with spectral widths of 31.82 and 14.06 ppm for the ^15^N and ^1^H dimensions, respectively, 256 (t_1_) and 2048 (t_2_) complex points, with 16 scans, and a recycle delay of 1 s. The experiments were repeated for 40 μM apo PKG I CNB-B, cGMP-bound, as well as the different cGMP analogs from the CHESCA perturbation library (i.e., Rp-cGMPs, Sp-cGMP, and 2′OMe-cGMP) at saturating concentration (>1 mM). In the case of T-CHESCA, the experiments were acquired at 296, 301, 306, 311, and 316 K. The samples were equilibrated for at least 15 min after every temperature change. Spectra processing was implemented through NMRPipe (*46*). Data analysis of T- and CL-CHESCA was performed with NMRFAM-Sparky (*42*). All the spectra for the chemical shift analysis were referenced to ^15^N-acetylglycine. The correlation matrices were built with Sparky-CHESCA as previously described (*42*), after assigning the different perturbation spectra (Apo, cGMP, Rp-cGMPs, Sp-cGMP, and 2′OMe-cGMP).

The cutoff thresholds for the ^15^N and ^1^H chemical shifts were established at 5 and 10 Hz, respectively. For the calculations of CCS, a scaling factor of 0.2 was applied to the ^15^N dimension. A Pearson’s correlation coefficient (*R*) threshold of 0.98 (T-CHESCA) and 0.97 (CL-CHESCA) was set, and CHESCA matrices were generated for all temperatures (T-CHESCA) using the same method. Only pairs of residues with computable *R* values across all five temperatures were included in the analysis. The “CHESCA-CL” feature within Sparky-CHESCA (*42*) was used to create the complete linkage clusters, with the correlation coefficient threshold for residues in these clusters set at 0.98 (T-CHESCA) and 0.97 (CL-CHESCA). In addition, ChimeraX was used to visualize the residues identified through T- and CL-CHESCA on the PKG Iβ structure (4KU8 and 4KU7).

## References

[1] F. M. Davis, D. L. Rateri, A. Daugherty, Mechanisms of aortic aneurysm formation: Translating preclinical studies into clinical therapies. Heart 100, 1498–1505 (2014). 25060754 10.1136/heartjnl-2014-305648

[2] D. M. Milewicz, S. K. Prakash, F. Ramirez, Therapeutics targeting drivers of thoracic aortic aneurysms and acute aortic dissections: Insights from predisposing genes and mouse models. Annu. Rev. Med. 68, 51–67 (2017). 28099082 10.1146/annurev-med-100415-022956 PMC5499376

[3] G. K. Schwaerzer, H. Kalyanaraman, D. E. Casteel, N. D. Dalton, Y. Gu, S. Lee, S. Zhuang, N. Wahwah, J. M. Schilling, H. H. Patel, Q. Zhang, A. Makino, D. M. Milewicz, K. L. Peterson, G. R. Boss, R. B. Pilz, Aortic pathology from protein kinase G activation is prevented by an antioxidant vitamin B12 analog. Nat. Commun. 10, 3533 (2019). 31387997 10.1038/s41467-019-11389-1 PMC6684604

[4] D. C. Guo, E. Regalado, D. E. Casteel, R. L. Santos-Cortez, L. Gong, J. J. Kim, S. Dyack, S. G. Horne, G. Chang, G. Jondeau, C. Boileau, J. S. Coselli, Z. Li, S. M. Leal, J. Shendure, M. J. Rieder, M. J. Bamshad, D. A. Nickerson, C. Kim, D. M. Milewicz, Recurrent gain-of-function mutation in PRKG1 causes thoracic aortic aneurysms and acute aortic dissections. Am. J. Hum. Genet. 93, 398–404 (2013). 23910461 10.1016/j.ajhg.2013.06.019 PMC3738837

[5] D. E. Casteel, E. V. Smith-Nguyen, B. Sankaran, S. H. Roh, R. B. Pilz, C. Kim, A crystal structure of the cyclic GMP-dependent protein kinase Iβ dimerization/docking domain reveals molecular details of isoform-specific anchoring. J. Biol. Chem. 285, 32684–32688 (2010). 20826808 10.1074/jbc.C110.161430 PMC2963381

[6] T. Münzel, R. Feil, A. Mülsch, S. M. Lohmann, F. Hofmann, U. Walter, Physiology and pathophysiology of vascular signaling controlled by cyclic guanosine 3′,5′-cyclic monophosphate-dependent protein kinase. Circulation 108, 2172–2183 (2003). 14597579 10.1161/01.CIR.0000094403.78467.C3

[7] A. Pfeifer, P. Ruth, W. Dostmann, M. Sausbier, P. Klatt, F. Hofmann, Structure and function of cGMP-dependent protein kinases. Rev. Physiol. Biochem. Pharmacol. 135, 105–149 (1999). 9932482 10.1007/BFb0033671

[8] R. Sharma, J. J. Kim, L. Qin, P. Henning, M. Akimoto, B. Vanschouwen, G. Kaur, B. Sankaran, K. R. Mackenzie, G. Melacini, D. E. Casteel, F. W. Herberg, C. Kim, An auto-inhibited state of protein kinase G and implications for selectiveactivation. eLife 11, e79530 (2022). 35929723 10.7554/eLife.79530 PMC9417419

[9] G. Y. Huang, J. J. Kim, A. S. Reger, R. Lorenz, E.-W. Moon, C. Zhao, D. E. Casteel, D. Bertinetti, B. Vanschouwen, R. Selvaratnam, J. W. Pflugrath, B. Sankaran, G. Melacini, F. W. Herberg, C. Kim, Structural basis for cyclic-nucleotide selectivity and cGMP-selective activation of PKG I. Structure 22, 116–124 (2014). 24239458 10.1016/j.str.2013.09.021 PMC4019043

[10] S. H. Francis, J. L. Busch, J. D. Corbin, cGMP-dependent protein kinases and cGMP phosphodiesterases in nitric oxide and cGMP action. Pharmacol. Rev. 62, 525–563 (2010). 20716671 10.1124/pr.110.002907 PMC2964902

[11] A. Buglioni, J. C. Burnett, New pharmacological strategies to increase cGMP. Annu. Rev. Med. 67, 229–243 (2016). 26473417 10.1146/annurev-med-052914-091923

[12] Q. Kong, R. M. Blanton, Protein kinase G I and heart failure: Shifting focus from vascular unloading to direct myocardial antiremodeling effects. Circ. Heart Fail. 6, 1268–1283 (2013). 24255056 10.1161/CIRCHEARTFAILURE.113.000575 PMC4213067

[13] J. Abramson, J. Adler, J. Dunger, R. Evans, T. Green, A. Pritzel, O. Ronneberger, L. Willmore, A. J. Ballard, J. Bambrick, S. W. Bodenstein, D. A. Evans, C. C. Hung, M. O’Neill, D. Reiman, K. Tunyasuvunakool, Z. Wu, A. Žemgulytė, E. Arvaniti, C. Beattie, O. Bertolli, A. Bridgland, A. Cherepanov, M. Congreve, A. I. Cowen-Rivers, A. Cowie, M. Figurnov, F. B. Fuchs, H. Gladman, R. Jain, Y. A. Khan, C. M. R. Low, K. Perlin, A. Potapenko, P. Savy, S. Singh, A. Stecula, A. Thillaisundaram, C. Tong, S. Yakneen, E. D. Zhong, M. Zielinski, A. Žídek, V. Bapst, P. Kohli, M. Jaderberg, D. Hassabis, J. M. Jumper, Accurate structure prediction of biomolecular interactions with AlphaFold 3. Nature 630, 493–500 (2024). 38718835 10.1038/s41586-024-07487-w PMC11168924

[14] S. H. Francis, I. V. Turko, J. D. Corbin, Cyclic nucleotide phosphodiesterases: Relating structure and function. Prog. Nucleic Acid Res. Mol. Biol. 65, 1–52 (2000). 10.1016/s0079-6603(00)65001-8 11008484

[15] M. E. Jost, M. Schweizer, P. Henning, C. Gorzelanny, M. Lehners, B. Ellinger, J. Boix-Campos, J.-M. Kux, S. Singh, A. Fachinger, K. Martinez Pomier, B. VanSchouwen, A. M. Billing, D. Biedenweg, M. Schweizer, S. Siegel, R. Reimer, M. Brandt, L. Priesmeier, U. Fuchs, J. Pflaumenbaum, V. O. Nikolaev, R. Newbury-Ecob, A. Wilsdon, M. Rybczynski, P. Gehle, L. C. Zelarayán, T. Stafforst, R. Feil, M. M. Rinschen, O. Pless, P. Eaton, P. J. Sáez, O. Otto, G. Melacini, T. J. Demal, T. Eschenhagen, F. W. Herberg, F. Cuello, Activating PRKG1 variant enhances smooth muscle cell deformability to cause aortopathy. JACC Basic Transl. Sci. 11, 101452 (2026). 41544301 10.1016/j.jacbts.2025.101452 PMC12834846

[16] A. P. Kornev, S. S. Taylor, L. F. Ten Eyck, A generalized allosteric mechanism for cis-regulated cyclic nucleotide binding domains. PLoS Comput. Biol. 4, e1000056 (2008). 18404204 10.1371/journal.pcbi.1000056 PMC2275311

[17] J. Wu, J. G. H. Bruystens, P. Sahoo, J. Bubis, R. A. Maillard, S. S. Taylor, R. Ilouz, N3A motifs in RIβ mediate allosteric crosstalk between cAMP and ATP in PKA activation. Protein Sci. 34, e70332 (2025). 41108566 10.1002/pro.70332 PMC12535202

[18] E. Butt, K. Abel, M. Krieger, D. Palm, V. Hoppe, J. Hoppe, U. Walter, cAMP- and cGMP-dependent protein kinase phosphorylation sites of the focal adhesion vasodilator-stimulated phosphoprotein (VASP) in vitro and in intact human platelets. J. Biol. Chem. 269, 14509–14517 (1994). 8182057

[19] A. Langer, P. A. Hampel, W. Kaiser, J. Knezevic, T. Welte, V. Villa, M. Maruyama, M. Svejda, S. Jähner, F. Fischer, R. Strasser, U. Rant, Protein analysis by time-resolved measurements with an electro-switchable DNA chip. Nat. Commun. 4, 2099 (2013). 23839273 10.1038/ncomms3099 PMC3719012

[20] B. VanSchouwen, R. Selvaratnam, R. Giri, R. Lorenz, F. W. Herberg, C. Kim, G. Melacini, Mechanism of cAMP partial agonism in protein kinase G (PKG). J. Biol. Chem. 290, 28631–28641 (2015). 26370085 10.1074/jbc.M115.685305 PMC4661378

[21] J. J. Kim, R. Lorenz, S. T. Arold, A. S. Reger, B. Sankaran, D. E. Casteel, F. W. Herberg, C. Kim, Crystal structure of PKG I:cGMP complex reveals a cGMP-mediated dimeric interface that facilitates cGMP-induced activation. Structure 24, 710–720 (2016). 27066748 10.1016/j.str.2016.03.009 PMC4856591

[22] J. J. Kim, D. E. Casteel, G. Huang, T. H. Kwon, R. K. Ren, P. Zwart, J. J. Headd, N. G. Brown, D. C. Chow, T. Palzkill, C. Kim, Co-crystal structures of PKG Iβ (92–227) with cGMP and cAMP reveal the molecular details of cyclic-nucleotide binding. PLOS ONE 6, e18413 (2011). 21526164 10.1371/journal.pone.0018413 PMC3080414

[23] F. Hofmann, H.-P. Gensheimer, C. Göbel, cGMP-dependent protein kinase. Eur. J. Biochem. 147, 361–365 (1985). 2982615 10.1111/j.1432-1033.1985.tb08758.x

[24] R. Selvaratnam, M. T. Mazhab-Jafari, R. Das, G. Melacini, The auto-inhibitory role of the EPAC hinge helix as mapped by NMR. PLOS ONE 7, e48707 (2012). 23185272 10.1371/journal.pone.0048707 PMC3504058

[25] R. Selvaratnam, B. VanSchouwen, F. Fogolari, M. T. Mazhab-Jafari, R. Das, G. Melacini, The projection analysis of NMR chemical shifts reveals extended EPAC autoinhibition determinants. Biophys. J. 102, 630–639 (2012). 22325287 10.1016/j.bpj.2011.12.030 PMC3274826

[26] R. Selvaratnam, S. Chowdhury, B. VanSchouwen, G. Melacini, Mapping allostery through the covariance analysis of NMR chemical shifts. Proc. Natl. acad. Sci. U.S.A. 108, 6133–6138 (2011). 21444788 10.1073/pnas.1017311108 PMC3076865

[27] S. Boulton, M. Akimoto, R. Selvaratnam, A. Bashiri, G. Melacini, A tool set to map allosteric networks through the NMR chemical shift covariance analysis. Sci. Rep. 4, 7306 (2014). 25482377 10.1038/srep07306 PMC4258684

[28] H. Mohamed, U. Baryar, A. Bashiri, R. Selvaratnam, B. VanSchouwen, G. Melacini, Identification of core allosteric sites through temperature- and nucleus-invariant chemical shift covariance. Biophys. J. 121, 2035–2045 (2022). 35538664 10.1016/j.bpj.2022.05.004 PMC9247469

[29] R. Das, V. Esposito, M. Abu-Abed, G. S. Anand, S. S. Taylor, G. Melacini, cAMP activation of PKA defines an ancient signaling mechanism. Proc. Natl. Acad. Sci. U.S.A 104, 93–98 (2007). 17182741 10.1073/pnas.0609033103 PMC1765484

[30] Y. Bai, J. J. Englander, L. Mayne, J. S. Milne, S. W. Englander, Thermodynamic parameters from hydrogen exchange measurements. Methods Enzymol. 259, 344–356 (1995). 8538461 10.1016/0076-6879(95)59051-x

[31] Y. Bai, T. R. Sosnick, L. Mayne, S. W. Englander, Protein folding intermediates: Native-state hydrogen exchange. Science 269, 193–197 (1995). 10.1126/science.7618079 PMC3432310 7618079

[32] C. E. Dempsey, Hydrogen exchange in peptides and proteins using NMR spectroscopy. Prog. Nucl. Magn. Reson. Spectrosc. 39, 135–170 (2001).

[33] Y. Bai, J. S. Milne, L. Mayne, S. W. Englander, Primary structure effects on peptide group hydrogen exchange. Proteins Struct. Funct. Bioinf. 17, 75–86 (1993). 10.1002/prot.340170110 PMC3438223 8234246

[34] Y. Bai, J. S. Milne, L. Mayne, S. W. Englander, Protein stability parameters measured by hydrogen exchange. Proteins Struct. Funct. Bioinf. 20, 4–14 (1994). 10.1002/prot.340200103 7824522

[35] D. M. Chu, J. D. Corbin, K. A. Grimes, S. H. Francis, Activation by cyclic GMP binding causes an apparent conformational change in cGMP-dependent protein kinase. J. Biol. Chem. 272, 31922–31928 (1997). 9395541 10.1074/jbc.272.50.31922

[36] M. E. Wall, S. H. Francis, J. D. Corbin, K. Grimes, R. Richie-Jannetta, J. Kotera, B. A. Macdonald, R. R. Gibson, J. Trewhella, Mechanisms associated with cGMP binding and activation of cGMP-dependent protein kinase. Proc. Natl. Acad. Sci. U.S.A. 100, 2380–2385 (2003). 12591946 10.1073/pnas.0534892100 PMC151349

[37] B. VanSchouwen, S. Boulton, G. Melacini, Mutual protein-ligand conformational selection drives cGMP vs. cAMP selectivity in protein kinase G. J. Mol. Biol. 433, 167202 (2021). 34400180 10.1016/j.jmb.2021.167202

[38] P. F. Cook, M. E. Neville, K. E. Vrana, F. Thomas Hartl, R. Roskoski, P. F. Cook, Adenosine cyclic 3′,5′-monophosphate dependent protein kinase: Kinetic mechanism for the bovine skeletal muscle catalytic subunit. Biochemistry 21, 5794–5799 (1982). 6295440 10.1021/bi00266a011

[39] G. B. Irvine, Determination of molecular size by size-exclusion chromatography (gel filtration). Curr. Protoc. Cell Biol. 6, 5.5.1–5.5.16 (2000). 10.1002/0471143030.cb0505s06 18228373

[40] D. Rodbard, A. Chrambach, Unified theory for gel electrophoresis and gel filtration. Proc. Natl. Acad. Sci. U.S.A. 65, 970–977 (1970). 4191703 10.1073/pnas.65.4.970 PMC283011

[41] J. D. Hunter, Matplotlib: A 2D graphics environment. Comput. Sci. Eng. 9, 90–95 (2007).

[42] H. Shao, S. Boulton, C. Olivieri, H. Mohamed, M. Akimoto, M. V. Subrahmanian, G. Veglia, J. L. Markley, G. Melacini, W. Lee, CHESPA/CHESCA-SPARKY: Automated NMR data analysis plugins for SPARKY to map protein allostery. Bioinformatics 37, 1176–1177 (2021). 32926121 10.1093/bioinformatics/btaa781 PMC8150123

[43] W. Lee, M. Rahimi, Y. Lee, A. Chiu, POKY: A software suite for multidimensional NMR and 3D structure calculation of biomolecules. Bioinformatics 37, 3041–3042 (2021). 33715003 10.1093/bioinformatics/btab180 PMC8479676

[44] S. Boulton, M. Akimoto, S. Akbarizadeh, G. Melacini, N. Allewell, Free energy landscape remodeling of the cardiac pacemaker channel explains the molecular basis of familial sinus bradycardia. J. Biol. Chem. 292, 6414–6428 (2017). 28174302 10.1074/jbc.M116.773697 PMC5391768

[45] R. Das, S. Chowdhury, M. T. Mazhab-Jafari, S. SilDas, R. Selvaratnam, G. Melacini, Dynamically driven ligand selectivity in cyclic nucleotide binding domains. J. Biol. Chem. 284, 23682–23696 (2009). 19403523 10.1074/jbc.M109.011700 PMC2749143

[46] F. Delaglio, S. Grzesiek, G. W. Vuister, G. Zhu, J. Pfeifer, A. Bax, NMRPipe: A multidimensional spectral processing system based on UNIX pipes. J. Biomol. NMR 6, 277–293 (1995). 8520220 10.1007/BF00197809

[47] N. Guex, M. C. Peitsch, SWISS-MODEL and the Swiss-PdbViewer: An environment for comparative protein modeling. Electrophoresis 18, 2714–2723 (1997). 9504803 10.1002/elps.1150181505

[48] A. Fiser, A. Sali, ModLoop: Automated modeling of loops in protein structures. Bioinformatics 19, 2500–2501 (2003). 14668246 10.1093/bioinformatics/btg362

[49] B. Vanschouwen, M. Akimoto, M. Sayadi, F. Fogolari, G. Melacini, Role of dynamics in the autoinhibition and activation of the hyperpolarization-activated cyclic nucleotide-modulated (HCN) ion channels. J. Biol. Chem. 290, 17642–17654 (2015). 25944904 10.1074/jbc.M115.651877 PMC4505013

[50] J. C. Phillips, R. Braun, W. Wang, J. Gumbart, E. Tajkhorshid, E. Villa, C. Chipot, R. D. Skeel, L. Kalé, K. Schulten, Scalable molecular dynamics with NAMD. J. Comput. Chem. 26, 1781–1802 (2005). 16222654 10.1002/jcc.20289 PMC2486339

[51] S. Boulton, K. Van, B. VanSchouwen, J. Augustine, M. Akimoto, G. Melacini, Allosteric mechanisms of nonadditive substituent contributions to protein-ligand binding. Biophys. J. 119, 1135–1146 (2020). 32882185 10.1016/j.bpj.2020.07.038 PMC7499118

[52] D. R. Roe, T. E. Cheatham, PTRAJ and CPPTRAJ: Software for processing and analysis of molecular dynamics trajectory data. J. Chem. Theory Comput. 9, 3084–3095 (2013). 26583988 10.1021/ct400341p

[53] D. A. Case, H. M. Aktulga, K. Belfon, D. S. Cerutti, G. A. Cisneros, V. W. D. Cruzeiro, N. Forouzesh, T. J. Giese, A. W. Götz, H. Gohlke, S. Izadi, K. Kasavajhala, M. C. Kaymak, E. King, T. Kurtzman, T. S. Lee, P. Li, J. Liu, T. Luchko, R. Luo, M. Manathunga, M. R. Machado, H. M. Nguyen, K. A. O’Hearn, A. V. Onufriev, F. Pan, S. Pantano, R. Qi, A. Rahnamoun, A. Risheh, S. Schott-Verdugo, A. Shajan, J. Swails, J. Wang, H. Wei, X. Wu, Y. Wu, S. Zhang, S. Zhao, Q. Zhu, T. E. Cheatham, D. R. Roe, A. Roitberg, C. Simmerling, D. M. York, M. C. Nagan, K. M. Merz, AmberTools. J. Chem. Inf. Model. 63, 6183–6191 (2023). 37805934 10.1021/acs.jcim.3c01153 PMC10598796

