# Supplementary material for: Aortic disease-linked mutations reveal unexpected convergent allosteric mechanisms of protein kinase G misregulation
Source: Sci Adv. 2026 Sep 2;12(36):eaeg1640. doi: 10.1126/sciadv.aeg1640 (PMC13537252; doi:10.1126/sciadv.aeg1640)
Supplement: Supplementary file 1 [file sciadv.aeg1640_sm.pdf]

Science Advances
AAAS

Supplementary Materials for

Aortic disease-linked mutations reveal unexpected convergent allosteric mechanisms of protein kinase G misregulation

Karla Martinez Pomier *et al.*

Corresponding author: Giuseppe Melacini, melacin@mcmaster.ca

Sci. Adv. **12**, eaeg1640 (2026)
DOI: 10.1126/sciadv.aeg1640

This PDF file includes:

Supplementary Text S1 and S2
Figs. S1 to S8
Tables S1 to S4

Supplementary text S1.

T-CHESCA *vs.* CLASS-CHESCA comparison

T-CHESCA and CLASS-CHESCA are complementary approaches that differ primarily in their balance between sensitivity and selectivity in detecting allosteric correlations. CLASS-CHESCA is a more selective method, which reduces false positives but may miss weaker or transient correlations, leading to potential false negatives. In contrast, conventional CHESCA may capture a broader set of correlations but may occasionally include spurious correlations or false positives. T-CHESCA provides a balanced compromise between these extremes by testing whether correlations between allosterically coupled residues are preserved across different temperatures. This approach allows for improved discrimination between meaningful allosteric couplings and incidental correlations, while maintaining sensitivity to relevant conformational changes. While T-CHESCA requires acquisition and assignment of NMR data across multiple temperatures, this added experimental effort enables a more robust identification of allosteric networks.

Supplementary text S2.

SwitchSENSE experiments

The Hill Fit Equation as used in the heliOS software version 2025.1.4

$$f(x) = \begin{cases} y_{start} & for\ x = 0 \\ y_{start} + \dfrac{x^N * (y_{end} - y_{start})}{K_D^N + x^N} & for\ x > 0 \end{cases}$$

y_{start} is the y-value of the curve when x equals zero, y_{end} y-value of the curve when x approaches positive infinity, K_D x- value of the curve's midpoint, i.e. the x-value at its median y-value, N Hill coefficient showing the degree of cooperativity in the binding system.

A

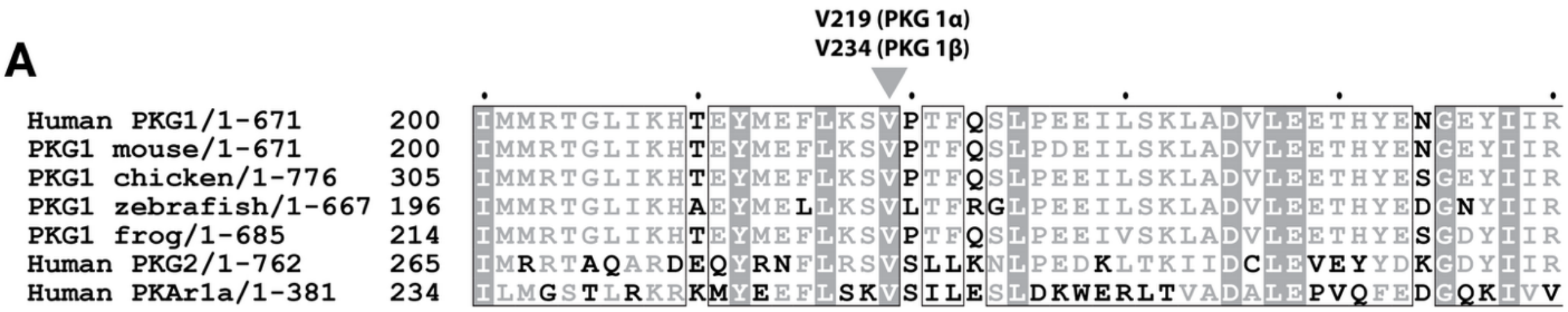

B

% Identity of Human PKG I with other model organisms

% identity	PKG (mouse)	PKG (Chicken)	PKG (Zebrafish)	PKG (Frog)	PKG2 (Human)	PKA r1a (Human)
Human PKG	99.55	96.87	88.91	89.04	53.04	32.91

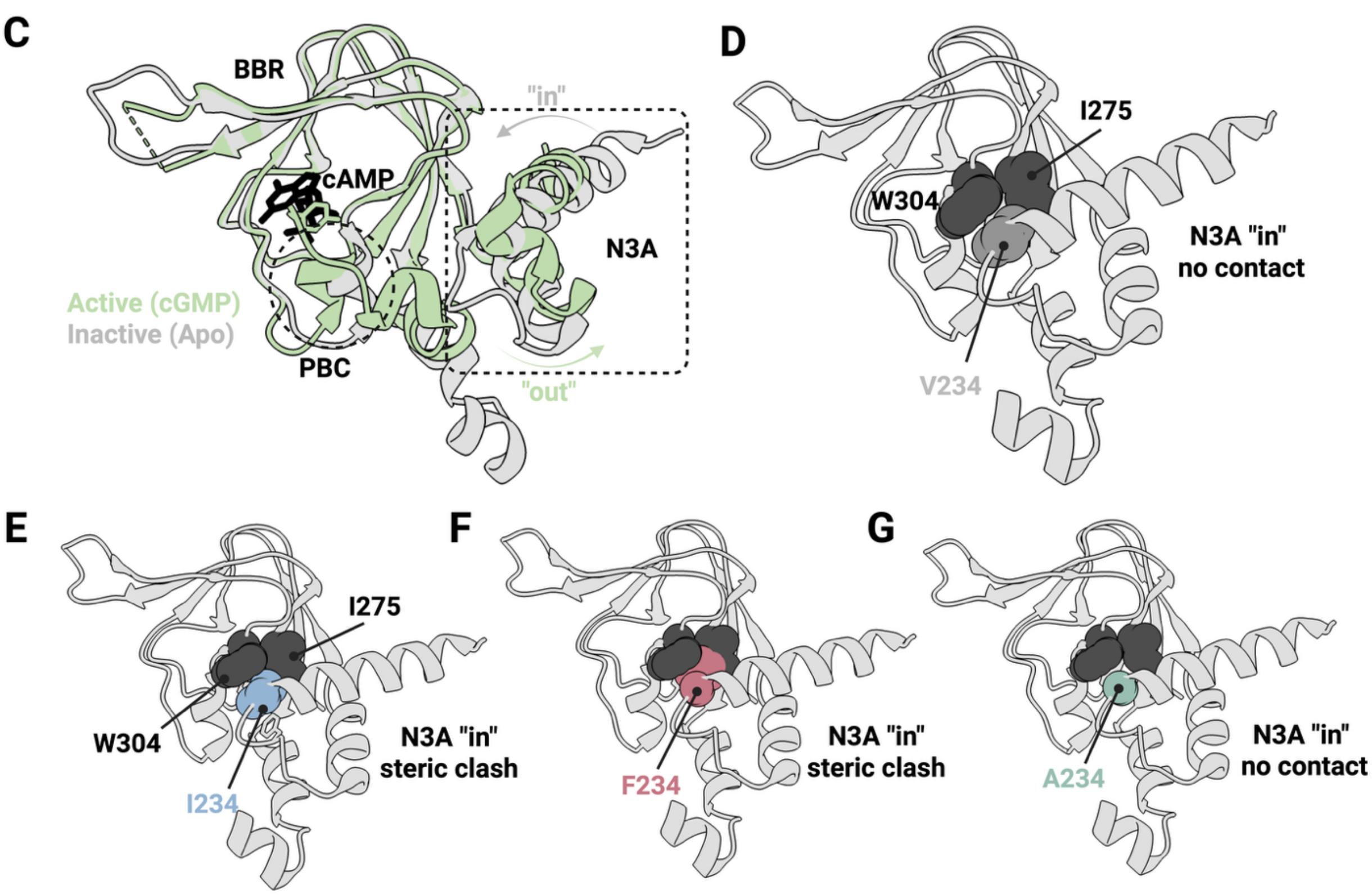

Fig. S1.
(**A**) Sequence alignment of human PKG Iα (200-300 or 215-315 in Iβ nomenclature) with different model organisms as well as human PKG II and Protein Kinase A regulatory subunit 1α (PKA R1a). (**B**) Percentage identity of PKG Iα/β (200-300 or 215-315) *vs*. other model organisms, PKG II and PKA R1a aligned regions. **(C)** Overlay of the crystal structures of the inactive (apo) and active (cGMP) conformations of PKG Iβ CNB-B (PDB codes: 4KU8 and 4KU7). In the inactive

conformation, the N3A adopts an “in” conformation; upon cGMP binding, the N3A adopts an “out” conformation. **(D)** In the apo conformation, V234 is close to residues W304 and I275 from the β-subdomain; however, there is no contact. **(E and F)** Variants V234I/F introduce bulkier hydrophobic residues that can cause a steric clash, possibly pushing the N3A to an out (active-like) conformation. (**G**) Variant V234A introduces a smaller side-chain with no expected contacts with neighbouring residues in the inactive/apo conformation.

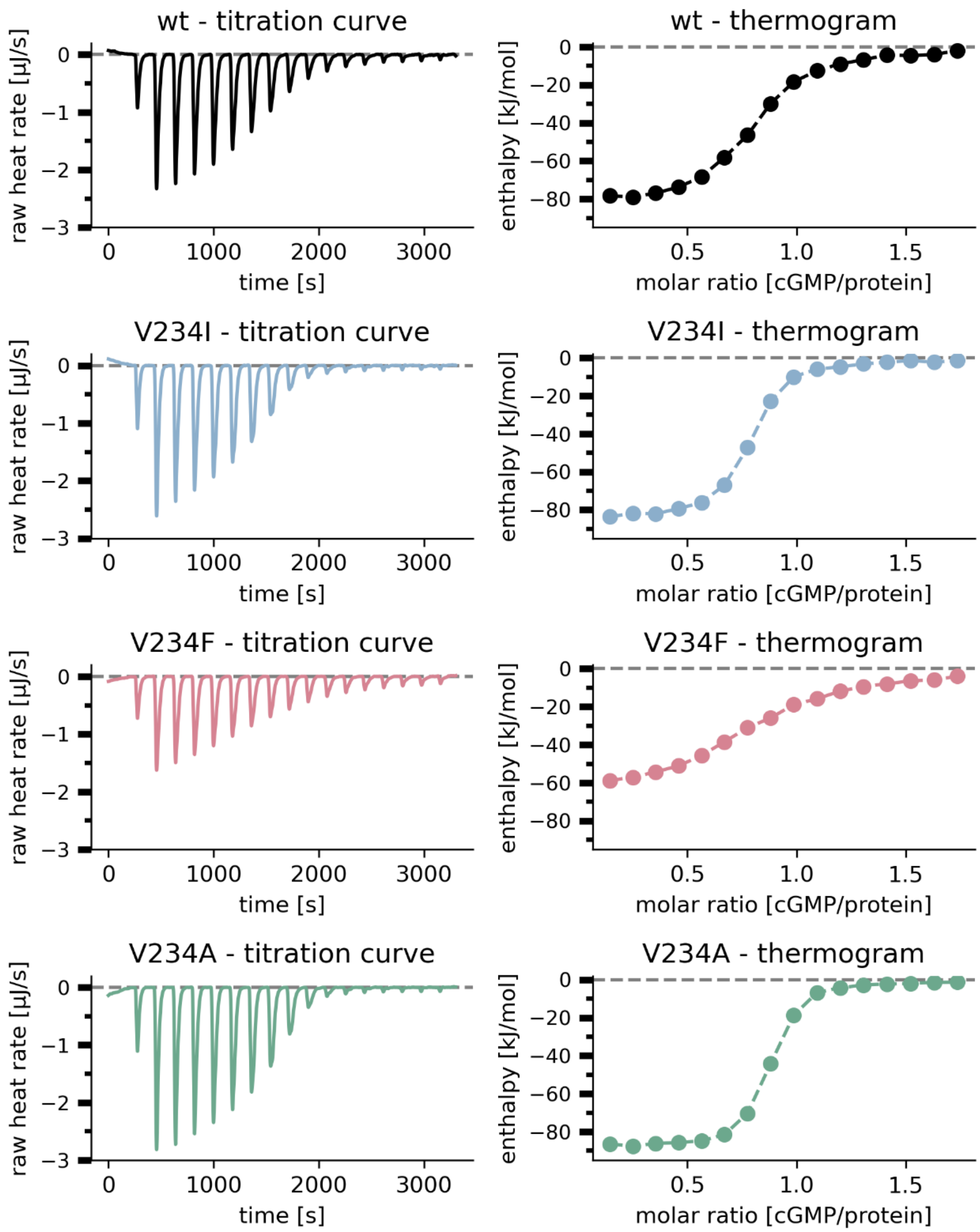

Fig. S2.

Isothermal titration calorimetry (ITC) analysis of cGMP binding to PKG I CNB-B (residues 219-369) wt and V234I, V34F, and V234A variants. Left panels show baseline-corrected raw heat flow traces for each titration, and right panels show the processed titration curves where the molar ratio of cGMP/protein is plotted against the enthalpy of the individual injections. The data shown here was recorded with the Affinity ITC SV device with 10 µM protein in the sample cell and 400 µM cGMP in the injection syringe.

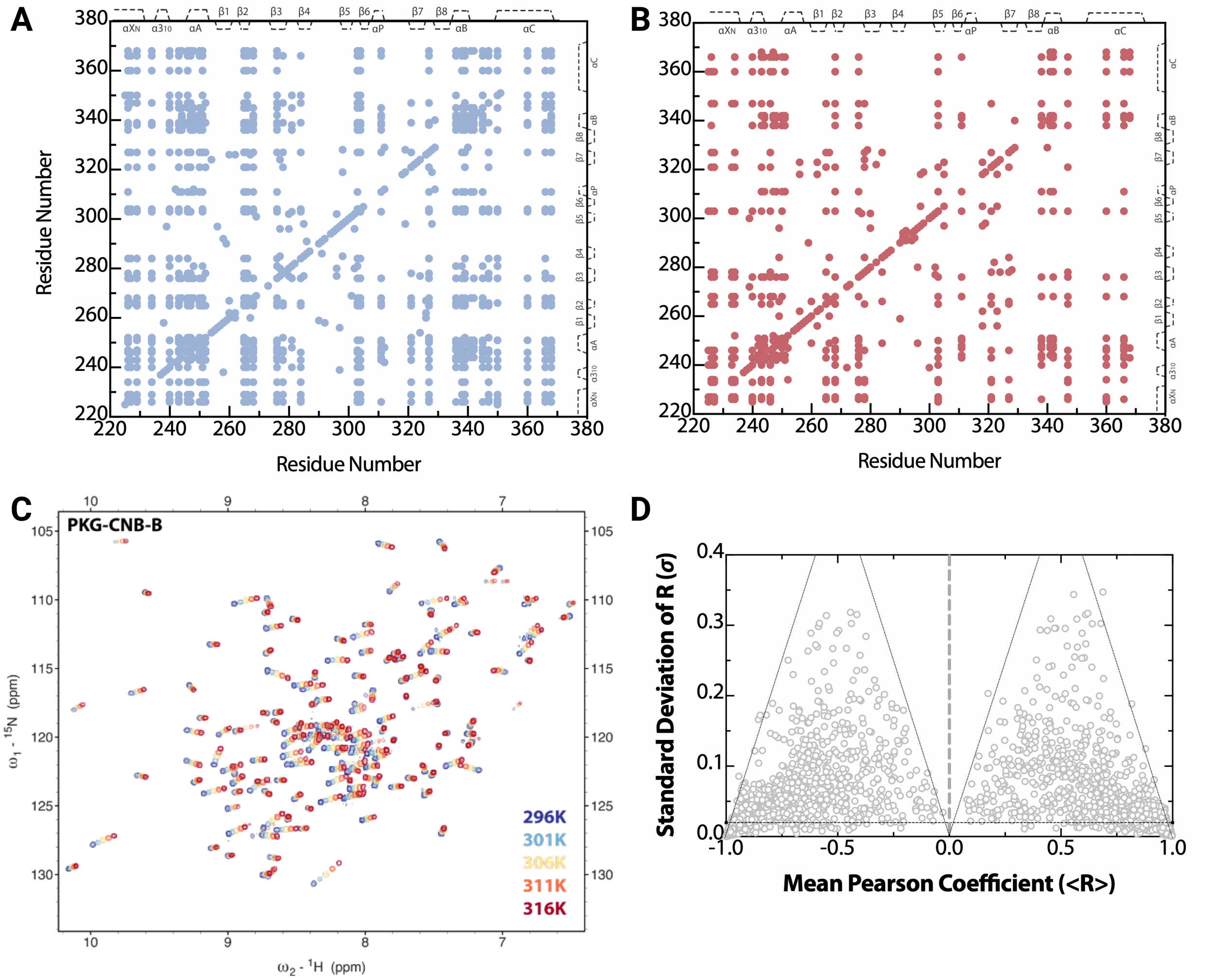

Fig. S3.

T-CHESCA analysis. (A- B) Chemical shift correlation (CHESCA) matrices for PKG I-CNB-B at **(A)** 296 K and **(B)** 316 K. **(C)** Overlay of unreferenced ^{1}H–^{15}N HSQC spectra acquired for the cGMP-bound (holo) PKG I-CNB-B at different temperatures. (**D)** The average correlation coefficient (<R>) for each residue pair was computed across five temperatures and plotted against its standard deviation (σ). Each circle represents a single residue pair.

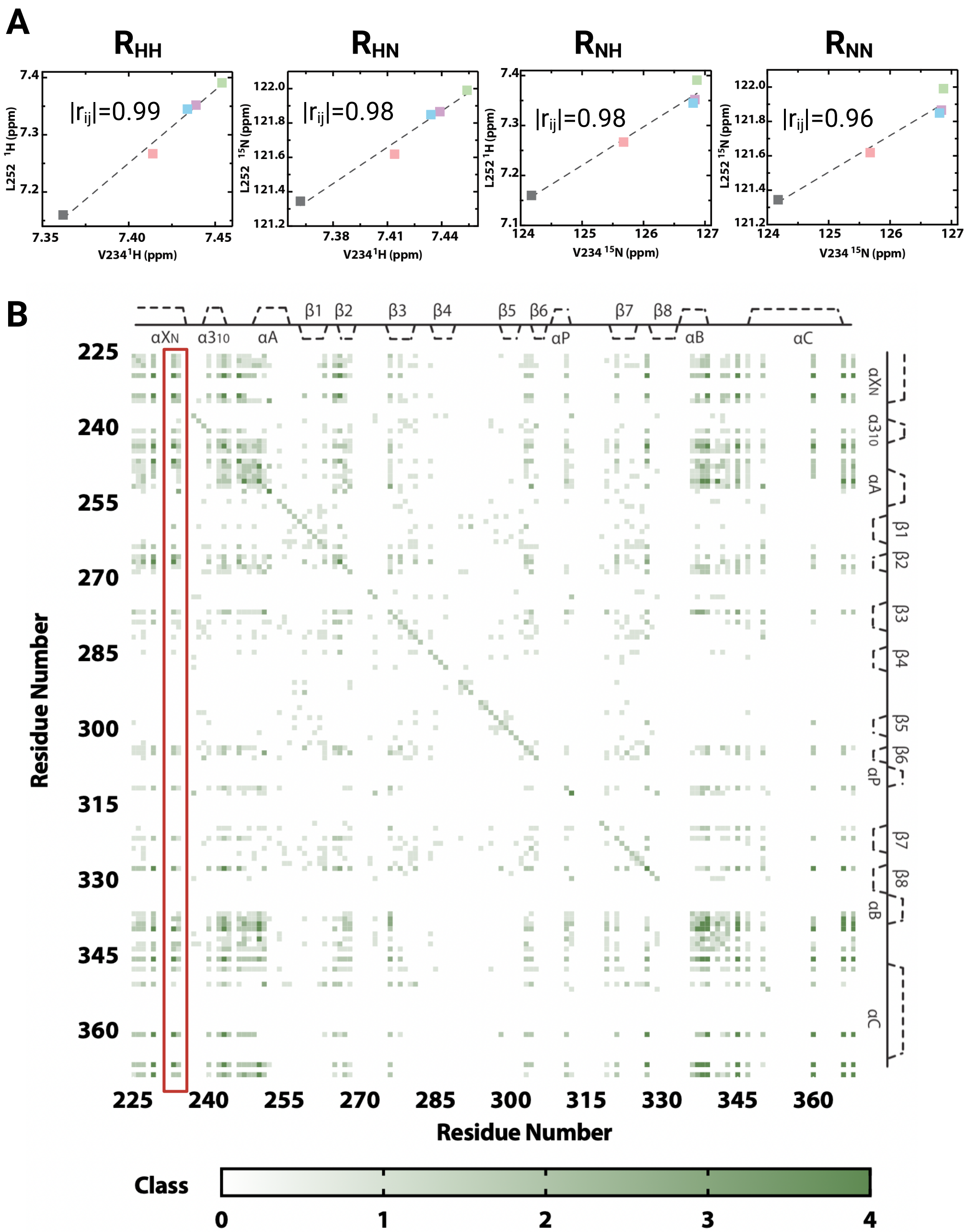

Fig. S4.

CLASS-CHESCA analysis. **(A)** Example of V234 and L252 different correlations (corresponding to R_{NH}–R_{NN}). The perturbation states are indicated by colour-coded squares, as in Fig. 7. **(B)** CLASS-CHESCA Matrix where the different colours represent the type of class that the residue pair correlation belongs to, as shown in the legend below. The region corresponding to V234 is enclosed in a red square.

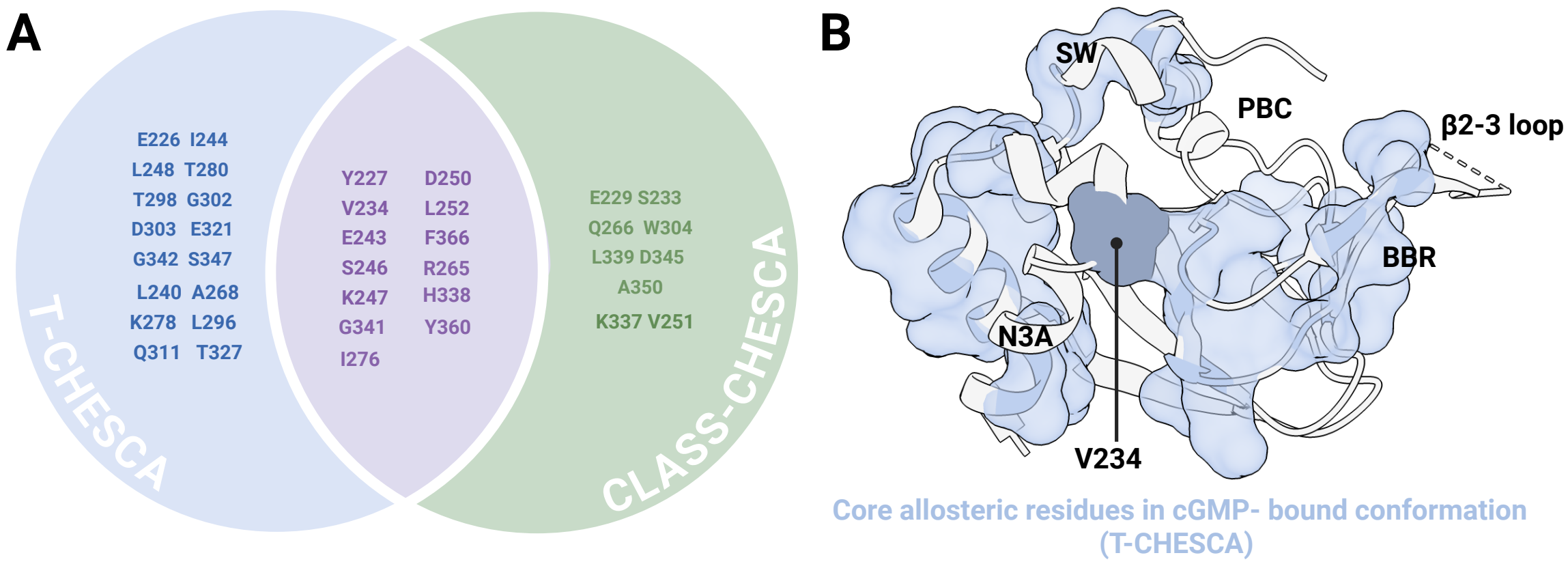

Fig. S5.
(**A**) Venn diagram showing allosteric core residues common to T- and CLASS-CHESCA. (**B**) T-CHESCA network mapped on the structure of PKG CNB-B bound to cGMP (PDB: 4KU7).

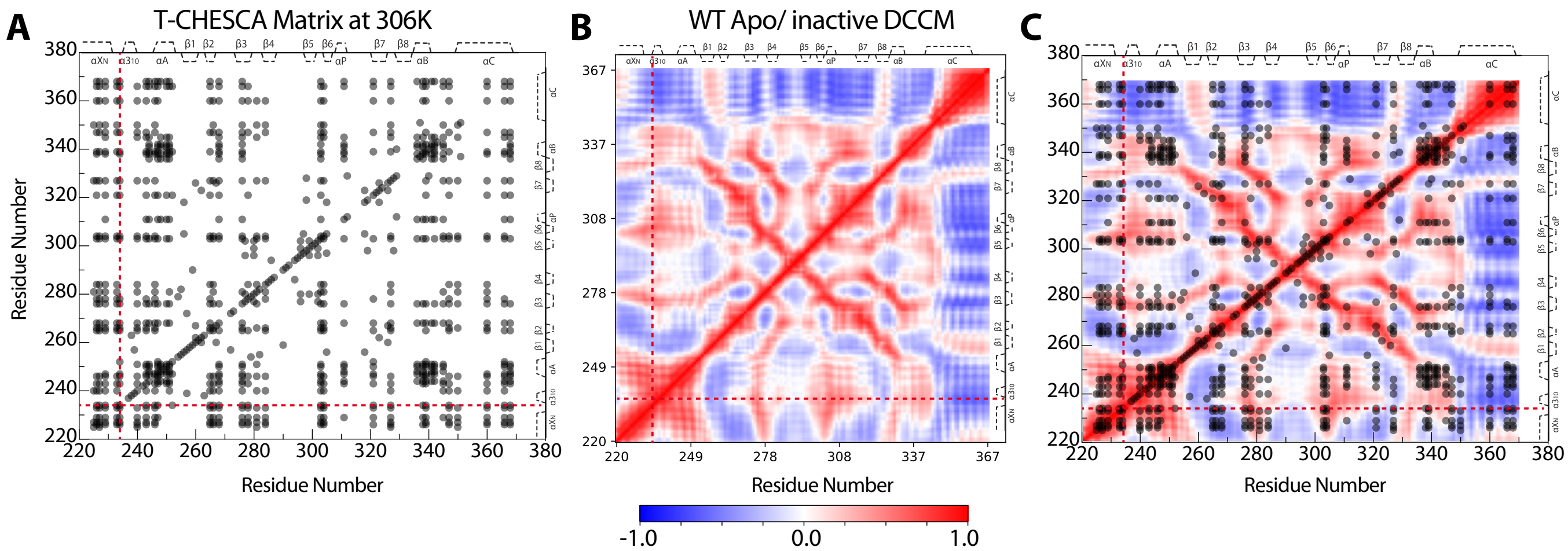

Fig. S6.

Integration of NMR-derived allosteric networks with MD simulations of PKG I CNB-B. (**A**) CHESCA matrix at 306 K. Dashed red lines indicate the V234 position in the N3A region. (**B**) Dynamic cross-correlation matrix (DCCM) derived from MD simulations showing correlated motions across the apo wt CNB-B domain. The color scale ranges from −1 to 1, where red indicates positively correlated motions, blue indicates anti-correlated motions, and white indicates weak or no correlation. Dashed red lines indicate the V234 position. (**C**) Overlay of the CHESCA and DCCM matrices.

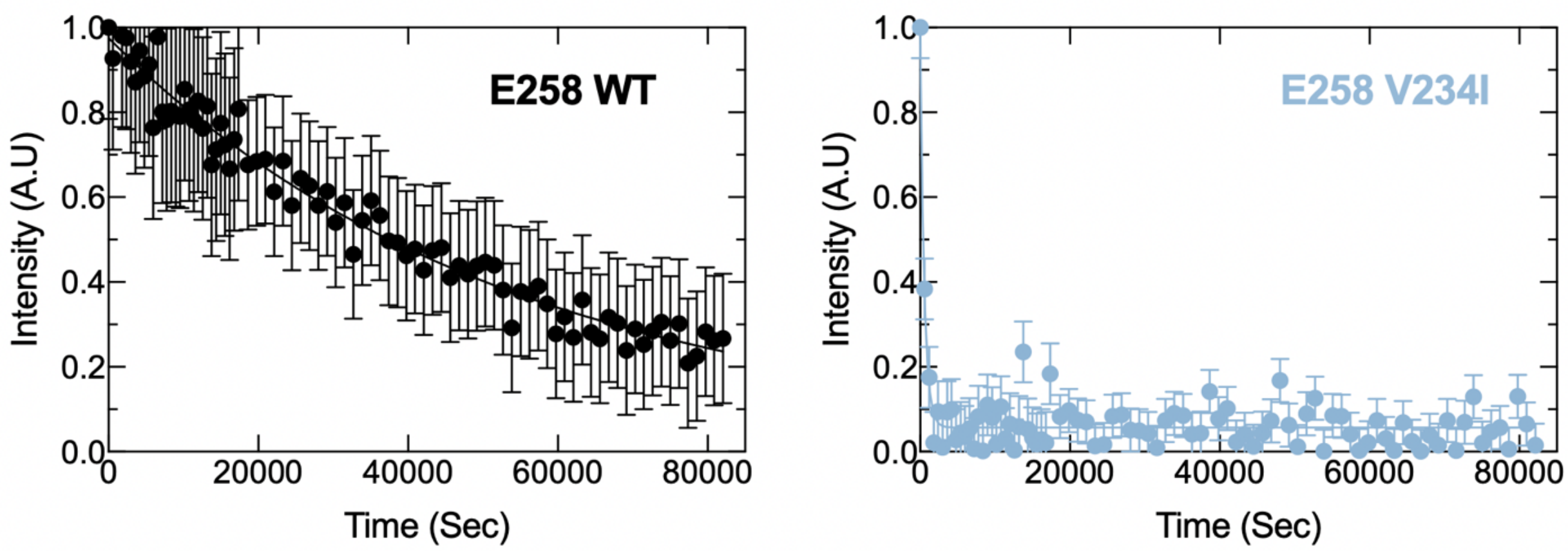

Fig. S7.
Hydrogen-deuterium exchange (HDX) rate decay of residue E258 in wt (black) and V234I (blue) PKG I-CNB-B, monitored by ^{1}H-^{15}N HSQC peak intensity as a function of time following exchange into D_2O. The curves were fitted using the CurveFit software by implementing Levenberg-Marquardt nonlinear least square exponential fitting. Error bars represent the uncertainty in peak intensity estimated from the spectral noise (signal-to-noise ratio) for each time point.

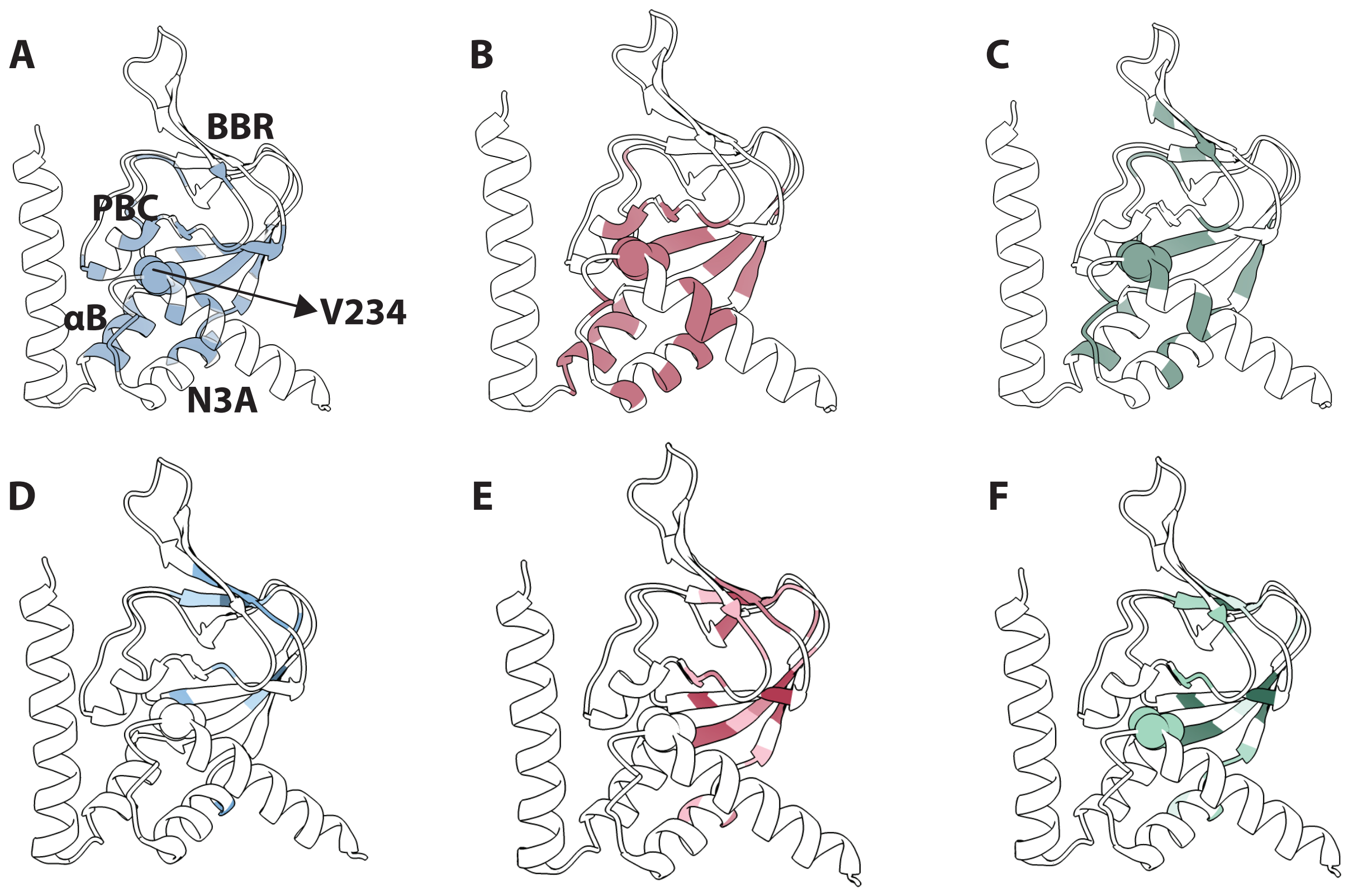

Fig. S8.

Comparative analyses of 3D maps for chemical shift perturbations (CSP) and HDX changes. (**A**) PKG I-CNB-B crystal structure (PDB:4KU8) highlighting the regions experiencing the most chemical shift perturbations (above average) for V234I (blue). Some key structural elements of domain B are labelled in the structure. (**B-C**) Same as (A) but for V234F (red, B) and V234A (green, C), respectively. (**D**) The residues whose protection factor (PF) was measured in the HDX experiment of V234I were plotted in the crystal structure of PKG I-CNB-B (PDB: 4KU8). The color code identifies residues experiencing the largest PF change (darker shade) to the least (lighter shade). (**E-F**) Same as (D) but for V234F (E) and V234A (F). This figure includes data previously shown in Fig. 5G–I and Fig. 8G–I, presented here for comparison.

Table S1. Kinase activity assay with the full-length proteins. Basal and maximal activities were determined using a microplate reader. Data are presented as means +- standard deviation. Activation constants with standard deviation. Significance was tested with an unpaired Student's t-test (n.s. = non-significant, *** = $p < 0.001$, **** = $p < 0.0001$).

**Protein**	**Maximal activity [U/mg]**	**Basal activity [U/mg]**	**K_{Act} [nM]**
**PKG Iβ WT**	13.4 ± 2.3<br>(n = 9)	0.8 ± 0.4<br>(n = 9)	180 ± 25<br>(n = 10)
**PKG Iβ V234I**	15.9 ± 3.4<br>(n = 9, n.s.)	1.3 ± 0.3<br>(n = 9, ***)	115 ± 25<br>(n = 8, ****)
**PKG Iβ V234F**	13.2 ± 1.9<br>(n = 11, n.s.)	1.2 ± 0.5<br>(n = 11, ****)	200 ± 25<br>(n = 10, n.s.)
**PKG Iβ V234A**	13.8 ± 1.6<br>(n = 11, n.s.)	1.0 ± 0.3<br>(n = 11, n.s.)	85 ± 20<br>(n = 8, ****)

Table S2. Size Exclusion Chromatography**.** Stokes´ radii determined via Size Exclusion Chromatography. Significance was tested with an unpaired Student's t-test (n.s. = non-significant, * = p < 0.05, ** = p< 0.01).

**Protein**	**Without cGMP [Å]**	**+25 μM cGMP [Å]**
**PKG Iβ WT**	60.5 ± 0.8<br>(n = 5)	66.9 ± 0.4<br>(n = 5)
**PKG Iβ V234I**	62.2 ± 0.9<br>(n = 5, **)	66.8 ± 0.7<br>(n = 5, n.s.)
**PKG Iβ V234F**	61.8 ± 0.8<br>(n = 5, *)	66.5 ± 0.6<br>(n = 5, n.s.)
**PKG Iβ V234A**	61.6 ± 0.7<br>(n = 6, *)	66.7± 0.6<br>(n = 5, n.s.)

Table S3. SwitchSENSE EC_{50} and Hill coefficients data for plots in Figure 3. Significance was tested with an unpaired Students t-test (n.s. = non-significant, * = $p< 0.05$, ** = $p< 0.01$, *** = $p< 0.001$).

	PKG Iβ wt	PKG Iβ V234I	PKG Iβ V234F	PKG Iβ V234A
EC_{50} [nM]	310 ± 65<br>(n=5)	195 ± 30<br>(n=6, **)	440 ± 100<br>(n=6, *)	125 ± 45<br>(n=6, ***)
n_{Hill}	1.57 ± 0.16<br>(n=5)	1.66 ± 0.26<br>(n=6, n.s.)	1.36 ± 0.17<br>(n=6, n.s.)	1.60 ± 0.09<br>(n=6, n.s.)

Table S4. Isothermal titration calorimetry. ITC data for the binding of cGMP to PKG I CNB-B. Significance was tested with an unpaired Students t-test (n.s. = non-significant, ** = p< 0.01, **** = p< 0.0001).

Protein (CNB-B)	K_D [nM]	ΔG [kJ/mol]	ΔH [kJ/mol]	-TΔS [kJ/mol]
**WT**	250 ± 20<br>(n =9)	-37.7 ± 0.2<br>(n =9)	-71.3 ± 7.5<br>(n =9)	33 ± 8<br>(n =9)
**V234I**	100 ± 10<br>(n = 9, ****)	-40.0 ± 0.2<br>(n = 9, ****)	-72.6 ± 7.4<br>(n = 9, n.s.)	33 ± 8<br>(n = 9, n.s.)
**V234F**	850 ± 65<br>(n = 8, ****)	-34.6 ± 0.2<br>(n = 8, ****)	-68.3 ± 4.3<br>(n = 8, n.s.)	34 ± 4<br>(n = 8, n.s.)
**V234A**	60 ± 10<br>(n = 6, ****)	-41.1 ± 0.3<br>(n = 6, ****)	-86.9 ± 6.6<br>(n = 6, **)	46 ± 7<br>(n = 6, **)
